# Synergistic retinal UCHL1 dysregulation and synaptic vulnerability reflect Alzheimer’s disease severity

**DOI:** 10.64898/2026.02.03.703406

**Published:** 2026-02-05

**Authors:** Altan Rentsendorj, Jean-Philippe Vit, Alexandre Hutton, Yosef Koronyo, Saba Shahin, Edward Robinson, Ernesto Barron, Anthony Rodriguez, Bhakta Prasad Gaire, Ousman Jallow, Julia Sheyn, Miyah Rene Davis, Alexander V. Ljubimov, Stuart L. Graham, Vivek K. Gupta, Mehdi Mirzaei, Debra Hawes, Keith L. Black, Lon S. Schneider, Alfredo A. Sadun, Jesse G. Meyer, Dieu-Trang Fuchs, Maya Koronyo-Hamaoui

**Affiliations:** 1 Department of Neurosurgery, Maxine Dunitz Neurosurgical Institute, Cedars-Sinai Medical Center, Los Angeles, CA, United States.; 2 Department of Computational Biomedicine, Cedars-Sinai Medical Center, Los Angeles, CA, United States.; 3 Doheny Eye institute, University of California Los Angeles, CA, United States.; 4 Board of Governors Regenerative Medicine Institute, Cedars-Sinai Medical Center, Los Angeles, CA, United States.; 5 David Geffen School of Medicine, University of California Los Angeles, Los Angeles, California, United States; 6 Department of Biomedical Sciences, Division of Applied Cell Biology and Physiology, Cedars-Sinai Medical Center, Los Angeles, CA, United States.; 7 Macquarie Medical School, Faculty of Medicine, Health and Human Sciences, Macquarie University, Sydney, NSW, Australia.; 8 ProHeme Diagnostics Pty Ltd, Sydney, NSW, Australia.; 9 Department of Pathology, Keck School of Medicine, University of Southern California, Los Angeles, CA, United States.; 10 Department of Psychiatry and The Behavioral Sciences, Department of Neurology, Keck School of Medicine, University of Southern California, Los Angeles, California, United States.; 11 Smidt heart institute, Cedars-Sinai Medical Center, Los Angeles CA, United States.; 12 Department of Neurology, Cedars-Sinai Medical Center, Los Angeles, CA, United States.

**Keywords:** Cognitive decline, Synaptic degeneration, Glutamatergic synapse, Amyloid-β_42_, Ubiquitin–proteasome system

## Abstract

Synaptic failure predicts cognitive decline in Alzheimer’s disease (AD), yet its impact and molecular drivers in the human retina remain unclear. Leveraging the retina as an accessible central nervous system (CNS) proxy, we integrated spatially resolved histopathology of retinal cross-sections with ultrastructural, proteomic, and biochemical profiling across independent postmortem cohorts spanning normal cognition, mild cognitive impairment due to AD (MCI), and AD dementia. We uncover early, progressive degeneration of excitatory glutamatergic synapses, evidenced by losses of presynaptic vesicular glutamate transporter 1 (VGLUT1) and synaptophysin, and postsynaptic density protein 95 (PSD95) and N-methyl-D-aspartate receptor subunit 2A (NMDAR2A), accompanied by disruption of synaptic ultrastructure. Retinal synaptic loss tightly associates with local accumulation of amyloid-β 42 (Aβ42) and immature tau species, heightened oxidative stress, and upregulation of the Aβ-binding death receptor p75 neurotrophin receptor (p75NTR). Notably, the synapse-enriched deubiquitinase ubiquitin C-terminal hydrolase L1 (UCHL1) is profoundly dysregulated, correlates with synaptic integrity and cognition, and emerges as the strongest retinal predictor of Braak stage and cognitive status in multivariable machine-learning models. Together, these findings position retinal Aβ/p75NTR-mediated UCHL1 imbalance as a proteostasis–synapse mechanistic hub and candidate biomarker reflecting AD severity.

## Introduction

Synaptic dysfunction is an early and defining process in the brains of Alzheimer’s disease (AD) patients and represents the most reliable predictor of cognitive decline^1–3^. Traditionally characterized by amyloid β-protein (Aβ) plaques and neurofibrillary tangles (NFTs) composed of hyperphosphorylated (p)tau protein^4–9^, AD is increasingly recognized as a disorder driven by early synaptic dysfunction, with deficits in glutamatergic signaling commonly preceding neuronal loss^2,10–17^. We previously found that overnight exposure to fibrillar, and moreover, oligomeric Aβ_42_ aggregates caused 50–70% loss of glutamatergic pre- and post-synaptic puncta in mouse primary cortical neuronal cultures^18^. However, despite decades of study, the mechanisms linking Aβ and tau accumulation to synaptic collapse and neurodegeneration remain unclear, underscoring the need for accessible biomarkers that faithfully reflect these pathological processes.

The retina, an embryological outgrowth of the brain, provides a uniquely accessible site into central nervous system (CNS) pathology. Increasing evidence demonstrates that AD hallmark pathologies—including Aβ deposits, tau inclusions, gliosis, and vascular changes—are detectable in the human retina and correlate with cerebral pathology and cognitive decline^19–39^. Advanced retinal imaging modalities, such as hyperspectral imaging and scanning laser ophthalmoscopy, have further detected Aβ and vascular dysfunction in living patients^27,40–51^. However, whether retinal synapses undergo degeneration that parallels brain neurodegeneration in AD, and which molecular mechanisms govern this process, remain largely unexplored.

One pathway strongly implicated in AD pathogenesis involves disruption of the ubiquitin–proteasome system (UPS), which contributes to Aβ and tau accumulation, oxidative stress, and synaptic loss^52–59^. Ubiquitin carboxyl-terminal hydrolase L1 (UCHL1), a neuron-enriched deubiquitinating enzyme and key component of the UPS, is essential for maintaining proteostasis, synaptic integrity, and neuronal survival^60–63^. In the brain, UCHL1 facilitates clearance of misfolded proteins and prevents aggregation of toxic Aβ42 and ptau^62,64–66^. Reduced UCHL1 expression in AD cortex and hippocampus correlates with synaptic loss and cognitive decline^56^. However, UCHL1 expression and its potential contribution to synaptic pathology have not been investigated in the AD retina. Another AD-relevant neurodegenerative pathway involves signaling via the p75 neurotrophin receptor (p75NTR). Initially characterized as a death receptor, p75NTR regulates axonal pruning, synaptic plasticity, and apoptosis in the CNS^67–71^. In brains from AD donors and rodent models, Aβ was shown to bind to p75NTR, driving neuritic degeneration, tau hyperphosphorylation, oxidative stress, and neuronal loss^72–77^. Furthermore, increased retinal p75NTR has been reported in experimental retinopathy models^78–80^; however, its relevance to AD retinal pathology and its potential relationship to synaptic failure remain unknown.

Here, we integrate spatially resolved histological quantification of supero- and infero-temporal (ST, IT) retinal cross-sections with ultrastructural and proteomic analyses to interrogate synaptic integrity, UPS function, and death receptor signaling across multiple independent human cohorts encompassing normal cognition (NC) controls, mild cognitive impairment (MCI due to AD), and AD dementia. We focus on excitatory glutamatergic synapses, quantifying presynaptic vesicular glutamate transporter 1 (VGLUT1) and synaptophysin (SYP), as well as postsynaptic postsynaptic density protein 95 (PSD95) and N-methyl-D-aspartate receptor subunit 2A (NMDAR2A). These proteins collectively orchestrate glutamate packaging and release, receptor-mediated excitatory transmission, and postsynaptic scaffolding, and their disruption is strongly implicated in cortical synaptic failure in AD^81–84^. Comprehensive proteomic, histological, biochemical, and transmission electron microscopy analyses reveal widespread degeneration of pre- and postsynaptic proteins, accompanied by ultrastructural collapse of synaptic architecture, early photoreceptor vulnerability, and marked dysregulation of UCHL1 in the retinas of MCI and AD patients. These changes strongly associate with retinal Aβ_42_ accumulation, immature tau species, oxidative stress, p75NTR signaling, and cognitive impairment, mirroring mechanisms described in the brain. Multivariable machine-learning models further identify UCHL1 as the strongest retinal predictor of Braak stage and cognitive status. Collectively, our findings establish the retina as a site of early synaptic and proteostatic collapse in AD and highlight retinal UCHL1 dysregulation as both a mechanistic hub and a candidate biomarker linking hallmark pathology to neuronal vulnerability.

## Results

**Figure 1.**
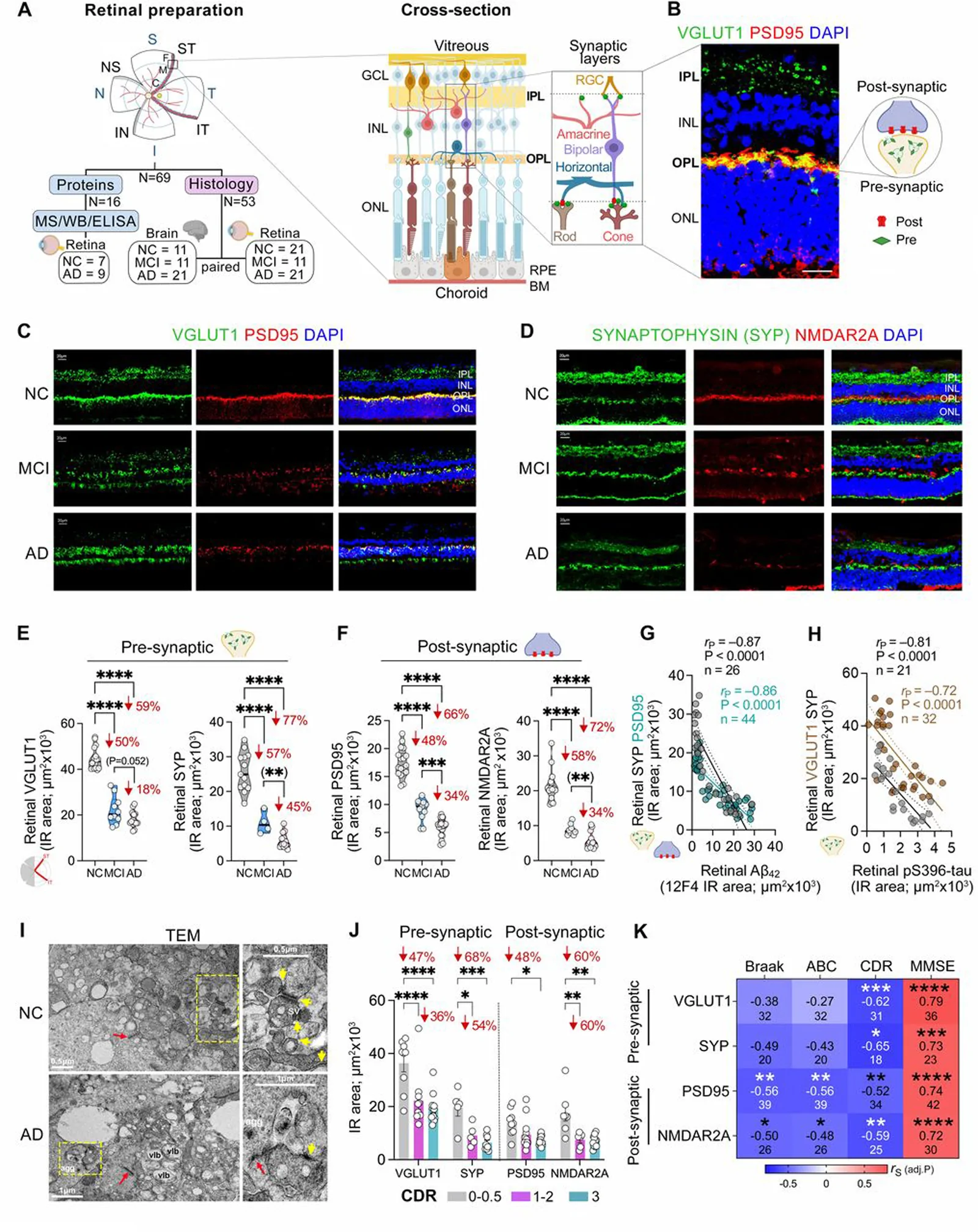
Synaptic loss in the retinas of MCI and AD patients and its relationship with core-AD pathologies and disease status. (**A**) Schematic of analyzed retinal cross-sections from individuals with normal cognition (NC), patients with mild cognitive impairment (MCI due to AD) or with Alzheimer’s disease (AD) in predefined geometrical regions within the superior- and inferior-temporal (ST/IT) retina (red strips), further subdivided into central (C), mid-periphery (M) and far-periphery (F) subregions. Retinal layers and basic synaptic architecture in the inner and outer plexiform layers (IPL and OPL), including pre- and post-synaptic terminals, are illustrated. The flow diagram outlines allocation of human donor eyes for histopathological and protein/ biochemical analyses, with subject numbers indicated. (**B**) Representative confocal image of retina immunolabeled for a pre- and post-synaptic marker, VGLUT1 (green) and PSD95 (red), showing strong synaptic labeling in the IPL and OPL. Nuclei are stained with DAPI (blue). Scale bar: 20 μm. (**C-D**) Representative micrographs/images of retinas from MCI, AD and NC donors, immunostained for pre-synaptic (VGLUT1 and synaptophysin SYP, green) and post-synaptic (PSD95 and NMDAR2A, red) markers. Nuclei are stained with DAPI (blue). Scale bars: 20 μm. (**E-F**) Violin plots showing quantitative immunoreactive (IR) area of retinal pre-synaptic (VGLUT1, SYP) and post-synaptic (PSD95, NMDAR2A) markers in MCI (n = 4–11) and AD (n = 12–20) patients, compared to age and sex-matched NC controls (n = 13–18). (**G-H**) Pearson’s correlation coefficient (*r*_P_) analysis showing a strong inverse relationship between (**G**) retinal PSD95, SYP and retinal Aβ_42_ (12F4) burden, and (**H**) retinal VGLUT1, SYP and retinal pS396-tau burden. (**I**) Transmission electron microscopy (TEM) images of OPL region from NC and AD retinas, labeled with pre- and post-synaptic markers, VGLUT1 and PSD95, respectively. Yellow arrowheads show synaptic clefts between pre- and post-synaptic terminals. Pre-synaptic terminals containing electron dense synaptic vesicles (SVs) are marked. Red arrows highlight membrane-docked pre-synaptic ribbons, which are preserved in NC retinas but are detached and floating in AD retinas. agg, electron dense aggregate; vlb, blebbing leakage. Scale bar: 0.5– 1 μm. (**J**) Quantitative analysis of retinal VGLUT1, SYP, PSD95 and NMDAR2A, stratified by clinical dementia rating (CDR) scores (CDR 0–0.5 = 5–9; CDR 1–2 = 5–12; CDR 3 = 8–13). (**K**) Heatmap showing Spearman’s correlation coefficients (*r*_S_) and significance (adjusted P values with Holm-Šídák multiple-comparison correction method for each retinal neuronal marker against 4 brain pathology and cognitive scores) between retinal synaptic markers and brain AD pathology (BRAAK, ABC scores) and cognitive performance/status (MMSE, CDR). Violin plots show individual subjects (circles) with lower, median and upper quartiles. Bar graph displays group means ± SEMs., *P < 0.05, ** P < 0.01, *** P < 0.001, **** P < 0.0001, by one-way ANOVA with Tukey’s post-hoc multiple comparison test. Percentage (%) changes are shown in red. IPL, inner plexiform layer; INL, inner nuclear layer; OPL, outer plexiform layer; ONL, outer nuclear layer. Schematic illustrations were created using Biorender.com.

To investigate synaptic integrity and associated neurodegeneration in postmortem retinas from patients with early and advanced-stage AD, we analyzed the spatiotemporal distribution and burden of synaptic proteins in superior- and inferior-temporal (ST/IT) retinal cross-sections from a cohort of 53 individuals comprising NC controls (n=21, mean age 82.6 ± 10.5 years, 11 females/10 males), MCI due-to-AD (n=11, mean age 89.3 ± 5.2 years, 7 females/4 males), and AD dementia (n=21, mean age 86.2 ± 8.9 years, 13 females/8 males). Age, sex, and post-mortem interval (PMI) did not differ significantly among the three diagnostic groups, and these variables were controlled for in each retinal biomarker subset, minimizing potential confounding effects. An additional cohort was used for mass spectrometry-based proteomics, Western Blot, and ELISA assays to assess dysregulation of synaptic and AD-related proteins in the temporal retina of AD patients and NC controls (n=16). Demographic, clinical, and neuropathological data for the cohorts used in histological and protein analyses is detailed in Figure 1A and Table 1 (individual human donors are listed in Table S1).

### Early and profound retinal synaptic degeneration strongly links to cognitive impairment in AD.

To determine the impact of AD on retinal synaptic integrity, we initially conducted a histological analysis of glutamatergic pre- (VGLUT1) and post- (PSD95) synaptic proteins in retinal synapse-rich inner and outer plexiform layers (IPL/OPL), in a cohort of AD and MCI patients compared with age- and sex-matched NC controls (n=51; Figure 1A-B). We further analyzed the presynaptic vesicle glycoprotein synaptophysin (SYP) and the postsynaptic glutamatergic receptor (NMDAR2A) in a subset of these subjects (n=29–36). Synaptic marker expression was next correlated with disease status in respective brain regions (n=43). Immunohistochemical (IHC) analysis of these pre- and post-synaptic markers showed early and progressive synaptic protein loss in MCI and AD retinas (Figure 1C and 1D; extended data in Figure S1A and S1B), with distinct distribution patterns for pre-synapses (IPL and OPL) and post-synapses (OPL dominant). Quantitative IHC analysis revealed highly significant reductions in pre-synaptic markers (VGLUT1 and SYP) by 50–57% in MCI retinas and 59–77% in AD retinas compared to NC controls (P < 0.0001, Figure 1E). Post-synaptic marker levels (PSD95 and NMDAR2A) followed similar trajectories, with marked decreases of 48–58% and 66–72% in MCI and AD retinas, respectively (P < 0.0001, Figure 1F). A detailed geometrical analysis of retinal synaptic markers across central (C), mid-peripheral (M), and far-peripheral (F) subregions demonstrated synaptic losses that were not uniformly distributed across the MCI/AD retina (Figure S1C-F). Indeed, the mid- and far-peripheral retina exhibited the most pronounced degeneration in AD, particularly for synaptophysin (Figure S1D). This spatial pattern aligns with prior evidence of peripheral-dominant retinal amyloidosis and tauopathy distribution^28,37^, suggesting localized synaptic vulnerability to AD pathology.

To test this, we evaluated the relationship between synaptic integrity and Aβ/tau pathology in the same retinal regions (representative images in Figure S1G and S1H); all multivariable correlations reported hereafter are presented as adjusted *P* values (P_*adj.*_). Pearson correlation coefficient (*r*_P_) analysis of retinal synaptic markers and retinal amyloidosis and tau isoforms (Table 2), revealed that all pre- and post-synaptic proteins, particularly synaptophysin and PSD95, were very closely associated with and highly vulnerable to Aβ_42_ levels in the same regions [*r* = –0.86-(–0.87), P_*adj.*_ < 0.0001; Figure 1G]. Synaptophysin was also tightly associated with Aβ oligomers in the retina (*r* = –0.83, P_*adj.*_ < 0.01; Table 2). With respect to retinal tauopathy, synaptophysin was very strongly correlated and susceptible to pathogenic tau species, particularly pS396-tau isoforms (*r* = –0.81, P_*adj.*_ < 0.0001; Figure 1H). In addition, retinal VGLUT1 levels were negatively correlated with Aβ_42_ and pS396-tau burden [*r* = –0.72-(–0.81), P_*adj.*_ < 0.0001; Table 2 and Figure 1H]. Most immature retinal tau species, including CitR_209_-tau, pS396-tau, and T22 tau oligomers, exhibited strong associations with both pre- and postsynaptic markers (Table 2), whereas AT8 p-tau showed significant correlations only with synaptophysin and PSD95 (*r* = –0.60-(–0.68), P_*adj.*_ < 0.05–0.01). Of note, mature forms of abnormal tau, including PHF-tau and MC1^+^ NFTs, did not correlate with any synaptic proteins in the retina (Table 2). These findings support a model in which retinal synaptic degeneration is driven by local Aβ and tau aggregation, mirroring well-characterized mechanisms in the AD brain. Interestingly, although retinal tauopathy colocalized with sites of synaptic loss in patients with MCI and AD, retinal Aβ_42_ was associated with the most detrimental impact on synaptic integrity.

Ultrastructural analysis of the IPL and OPL layers using transmission electron microscopy (TEM), following immunolabeling with pre-synaptic (VGLUT1) and post-synaptic (PSD95) markers, further confirmed synaptic morphological disruption and loss of synaptic integrity in the AD retina (Figures 1I). In NC control retinas, synapses displayed well-preserved architecture (yellow arrows), with intact clefts, membrane-anchored pre-synaptic ribbons (red arrows), and pre-synaptic terminals densely packed with electron-dense synaptic vesicles (SVs) aligned with clearly defined post-synaptic terminals (Figure 1I, upper panel). In contrast, AD retinas exhibited reduced synaptic density or strength, as well as severe ultrastructural synaptic abnormalities, including darkened and electron-dense aggregates (agg), fragmented and mis-localized free floating pre-synaptic ribbons within the cytoplasm, meaning, there was a loss of membrane-anchored ribbon structures, vesicular leakage, and cellular blebbing (vlb) (Figure 1I, lower panel). These morphological abnormalities are consistent with advanced synaptic degeneration and loss of structural integrity.

Next, we determined whether retinal synaptic loss reflected overall AD severity and stage, as indexed by brain pathology, including Braak stage and ABC scores, and cognitive status as assessed by the clinical dementia rating (CDR) and the mini-mental state examination (MMSE; Figure 1J and 1K; extended data in Figure S1I and S1J, and Tables S2-S5). Stratification by CDR categories revealed progressive reductions in synaptic markers, VGLUT1 by 36–47%, synaptophysin by 54–68%, PSD95 by 48%, and NMDAR2A by 60% in individuals with CDR 1–2 or CDR 3 as compared to individuals with CDR 0 (Figure 1J and Table S2). Stratification of our cohort per Braak stages, revealed similar patterns of significant reductions in retinal VGLUT1 (34–41%) and SYP (50–63%) in individuals with Braak stage III–VI compared to stage 0-II, whereas retinal PSD95 and NMDAR2A were significantly reduced by 52–54% only in individuals with stage V–VI but not III-IV (Figure S1J and Table S3). Individuals with MMSE scores of ≤26 demonstrated 52–67% reductions in retinal pre- and post-synaptic markers as compared to those with MMSE scores below 26 (Figure S1K and Table S4). Spearman’s rank pairwise correlation analyses revealed significant associations between these retinal synaptic markers and brain AD-related pathology, Aβ-plaque or NFT burden (Figure 1K and Table S5). While weak to moderate associations were found between retinal synaptic markers and brain Aβ-plaque or NFT neuropathological burden, a strong association was detected between retinal synaptophysin and brain NFT severity (*r* = –0.65, P < 0.01; Table S5). Notably, retinal post-synaptic markers (PSD95, NMDAR2A) compared with the pre-synaptic proteins, showed more significant correlations with disease stage (Braak, ABC), whereases the pre-synaptic markers (VGLUT1, SYP) exhibited slightly stronger associations with the cognitive deficit (CDR, MMSE; Figure 1K). Overall, our findings reveal robust, early, and spatially distinct patterns of synaptic density losses in the human AD retina which were tightly coupled to both retinal and brain pathology. The peripheral retina emerges as a focal point of synaptic vulnerability, likely reflecting early Aβ and tau accumulation. These results support the concept that the retina undergoes early synaptic degeneration prior to neuronal cell loss (neurodegeneration), parallelling disease processes in the brain.

### Proteomic evidence of retinal neurosynaptic deficits in AD with p75NTR upregulation.

We next sought to delineate synaptic- and neurodegeneration-targeted molecular pathways in the AD retina by analyzing an independent human cohort using an integrated protein biochemistry workflow encompassing Western blotting (WB), secondary mass spectrometry (MS), and quantitative enzyme-linked immunosorbent assay (ELISA). This cohort comprised neuropathologically confirmed AD patients (n = 9; mean age = 82.0 ± 15.7 years; 6 females, 3 males) and age- and sex-matched individuals with normal cognition (NC; n = 7; mean age = 76.7 ± 5.4 years; 4 females, 3 males). Initial proteomic findings from this cohort were previously reported (PRIDE ID: PXD040225)^28^. Preranked gene set enrichment analysis (GSEA)^85^ of AD retinal MS data further confirmed inhibited synapse- and vision-linked pathways, including KEGG ‘adherens junction’ and ‘phototransduction’, alongside GO ‘response to light stimulus’ and ‘actin filament–based transport,’ while activated GO ‘negative regulation of cell cycle’—consistent with disrupted retinal connectivity, impaired neuronal homeostasis, and neurodegeneration in AD (Figure S2).

**Figure 2.**
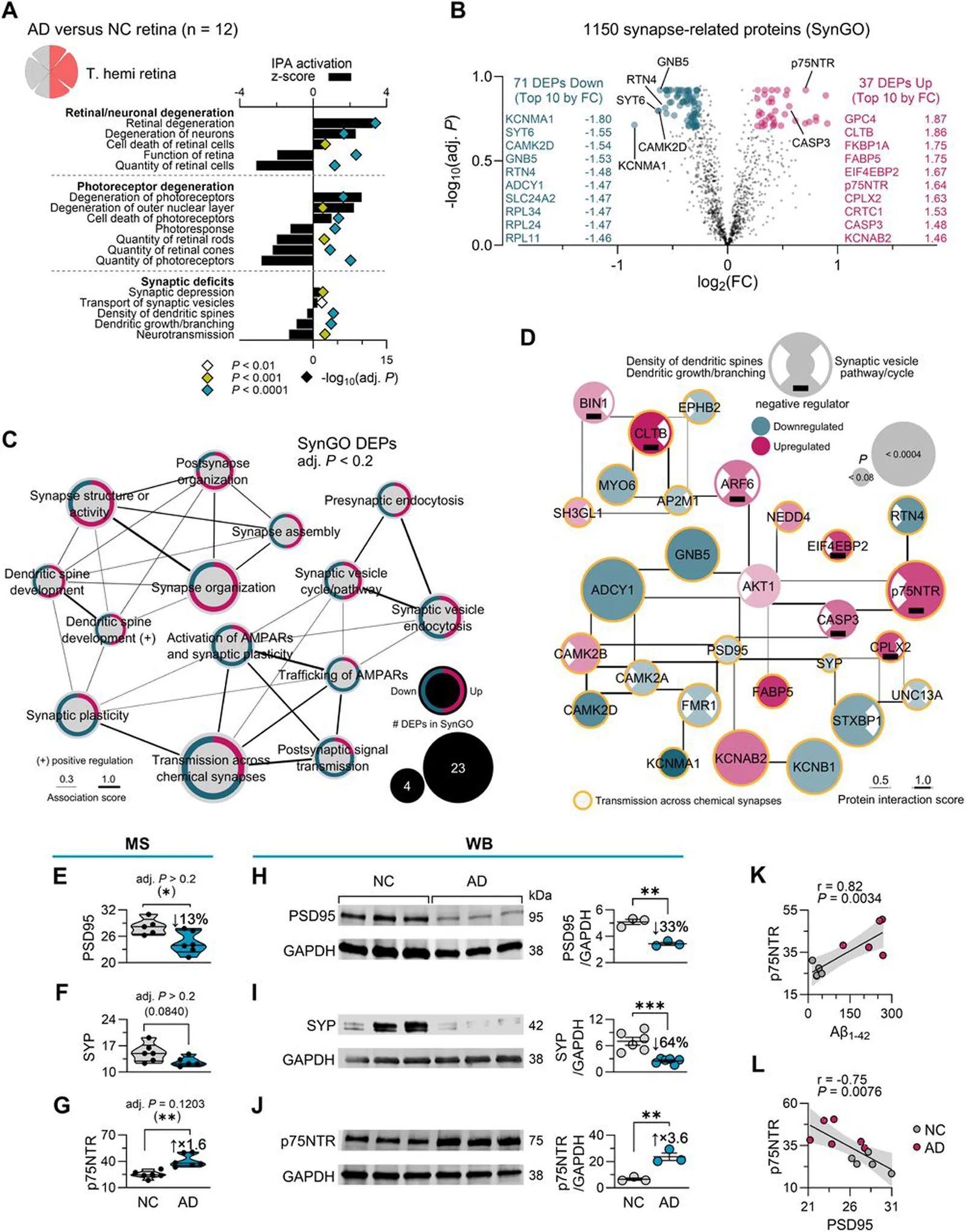
Dysregulated AD retinal proteome associated with synaptic and neuronal loss. (**A-F**) Pathway analysis of global proteomics via mass spectrometry (MS) in AD (n = 6) versus NC control (n = 6) retinas. For this analysis, DEPs were defined by |FC| > 1.2 and adj. *P* < 0.2. (**A**) Dysregulated biological pathways related to retinal/neuronal degeneration, photoreceptor degeneration and synaptic deficits, revealed by Ingenuity pathway analysis (IPA) of DEPs in the AD retina. Bar and symbol graphs represent activation z-scores, and Benjamini-Hochberg adjusted P-values, respectively. Range of P-values are indicated as color-coded symbols. (**B**) Volcano plot of dysregulated synapse-associated proteins in AD versus NC control retinas, extracted from SynGo release 1.2 knowledge base. The top 10 upregulated and downregulated DEPs are listed with their fold change (FC). (**C**) Pathway network (Metascape) from Gene Ontology (GO) analysis of synaptic-relevant DEPs revealed by SynGo. The size of the nodes represents the number of DEPs in SynGo, while the thickness of edges represents the overlapping DEPs (association score) between GO terms. (**D**) Interaction network analysis of DEPs related to synaptic plasticity, synaptic vesicle cycle and neurotransmission built from String v12.0 database. (**E-G**) Violin plots of the post-synaptic marker PSD95 (**E**), the pre-synaptic protein SYP (**F**) and the neuron-type-specific death receptor NGFR/p75NTR (**G**), quantified by MS, in AD versus control retinas. (**H-J**) Western blot analysis and normalized quantitation of retinal PSD95 (**H**), SYP (**I**) and p75NTR (**J**). (**K**) Pearson’s correlations between retinal Aβ_1–42_ levels, measured by ELISA, and retinal p75NTR, quantified by MS. (**L**) Pearson’s correlation between retinal PSD95 and retinal p75NTR. Violin plots show individual subjects (circles) with lower, median and upper quartiles. *P < 0.05, ** P < 0.01, *** P < 0.001, by unpaired Student t-test.

Further, Ingenuity Pathway Analysis (IPA) of differentially expressed proteins (DEPs: |fold change| > 1.2 and FDR-adjusted P values < 0.2) in AD versus NC retinas revealed perturbation of retinal homeostasis and function, with prominent photoreceptor degeneration, alongside synaptic dysfunction spanning synaptic depression, dendritic spine density/architecture, synaptic vesicle trafficking, and neurotransmission (Figure 2A). A substantial fraction of retinal proteins expressed in photoreceptors, including several with exclusive photoreceptor expression, was markedly dysregulated in AD (Figure S3A–B). Within photoreceptor outer segments, the site of phototransduction, opsins including rhodopsin (RHO), long-wave–sensitive opsin 1 (OPN1LW), and short-wave–sensitive opsin 1 (OPN1SW) were reduced by ~47%–61% in AD retinas, accompanied by significant decreases in cyclic nucleotide–gated channel subunits (CNGA1, CNGB1), transducin γ subunit (GNGT1), and Na^+^/Ca^2+^–potassium channels (SLC24A1/2), key components of the visual transduction cascade. Photoreceptor inner-segment proteins implicated in excitability and phototransduction—including the potassium channels KCNB1, KCNV2, and KCNMA1—were similarly decreased, together with peripherin-2 (PRPH2), in the AD retina (Figure S3A–B). In parallel, pro-apoptotic mediators were increased in the AD retina, including Fas associated protein with death domain (FADD), pro-apoptotic caspase 3 (CASP3) and the Aβ-binding death receptor p75-neurotrophin nerve growth factor receptor (p75NTR, also known as NGFR; Figure S3C–D)^76,86,87^. Retinal p75NTR levels tightly and inversely correlated with all photoreceptor markers (Figure S3E; *P*_*adj.*_ < 0.05), consistent with pronounced photoreceptor degeneration in AD.

Alongside neurodegeneration, synaptic integrity was compromised, as evidenced by downregulation of synaptic proteins enriched in photoreceptor terminals, including synaptotagmin-6 (SYT6) and retinoschisin-1 (RS1) (Figure S3A). We analyzed synapse-associated DEPs in AD retinas using the SynGo 1.2 database (Figure 2B–D). Of the 1602 synapse-associated proteins included in the database, 1150 were detected in the human retina, with 37 significantly upregulated and 71 significantly downregulated in the AD retina (Figure 2B; list of synaptic-associated DEPs in Tables S6 and S7). These accounted for approximately 14% and 15% of upregulated and downregulated DEPs in the AD retina, respectively (Figure S3F). Gene Ontology (GO)-guided pathway analysis of the 108 significantly altered proteins, queried across GO Biological Process, Reactome, KEGG, and WikiPathways, converged on synaptic dysregulation in AD retinas, marked by reduced synaptic plasticity, chemical synaptic and postsynaptic signaling, and AMPAR activation/trafficking, alongside increased signatures of synaptic organization and structural remodeling (Figure 2C). Consistent with these changes, positive regulators of synaptic transmission were largely downregulated, including the Ca^2^ /calmodulin-regulated adenylyl cyclase 1 (ADCY1/AC1) linked to visual/sensory and photoreceptor biology, the core G-protein subunit β5 (GNB5) which tunes synaptic signaling and light responses, and the synaptic markers PSD95 and SYP (Figure 2D). Conversely, negative regulators of synaptic vesicle recycling—including synaptic vesicle endocytosis-related clathrin light chain B (CLTB), bridging integrator 1 (BIN1) and ADP ribosylation factor 6 (ARF6)—and dendritic spine formation—including the translation initiation repressor eukaryotic translation initiation factor 4E binding protein 2 (EIF4EBP2), CASP3 and p75NTR—were upregulated in the AD retina (Figure 2D).

Western blotting of retinal homogenates from a subset of AD patients and NC individuals corroborated the MS findings, confirming reduced expression of PSD95 (33% decrease; *P*<0.01) and synaptophysin (SYP; 64% decrease; P<0.001), alongside an increase in p75NTR (3.6-fold; *P*<0.01) in AD retinas (Figure 2E–J; full extended blots in Figure S4). Given that Aβ_42_ binds p75NTR and can promote neuritic degeneration, synaptic loss, and apoptosis, we next tested the p75NTR association with Aβ_1–42_ peptide levels (measured by a quantitative sandwich ELISA) and synaptic markers (Figure 2K–L). Retinal p75NTR expression very strongly correlated with retinal Aβ_1–42_ levels (Figure 2K; r=0.82, *P*=0.0034) and was inversely associated with the glutamatergic postsynaptic marker PSD95 (Figure 2L; r=−0.75, P=0.0076). Together, these relationships support the existence of an Aβ_42_–p75NTR axis contributing to synaptic loss and neurodegeneration in the AD retina.

Overall, these proteomic and biochemical analyses demonstrate that AD retinas undergo profound photoreceptor marker loss and synaptic dysfunction, potentially driven by Aβ_42_ accumulation and p75NTR-mediated apoptosis.

### Oxidative stress and p75NTR apoptotic signaling-mediated synaptic loss in the AD retina.

**Figure 3.**
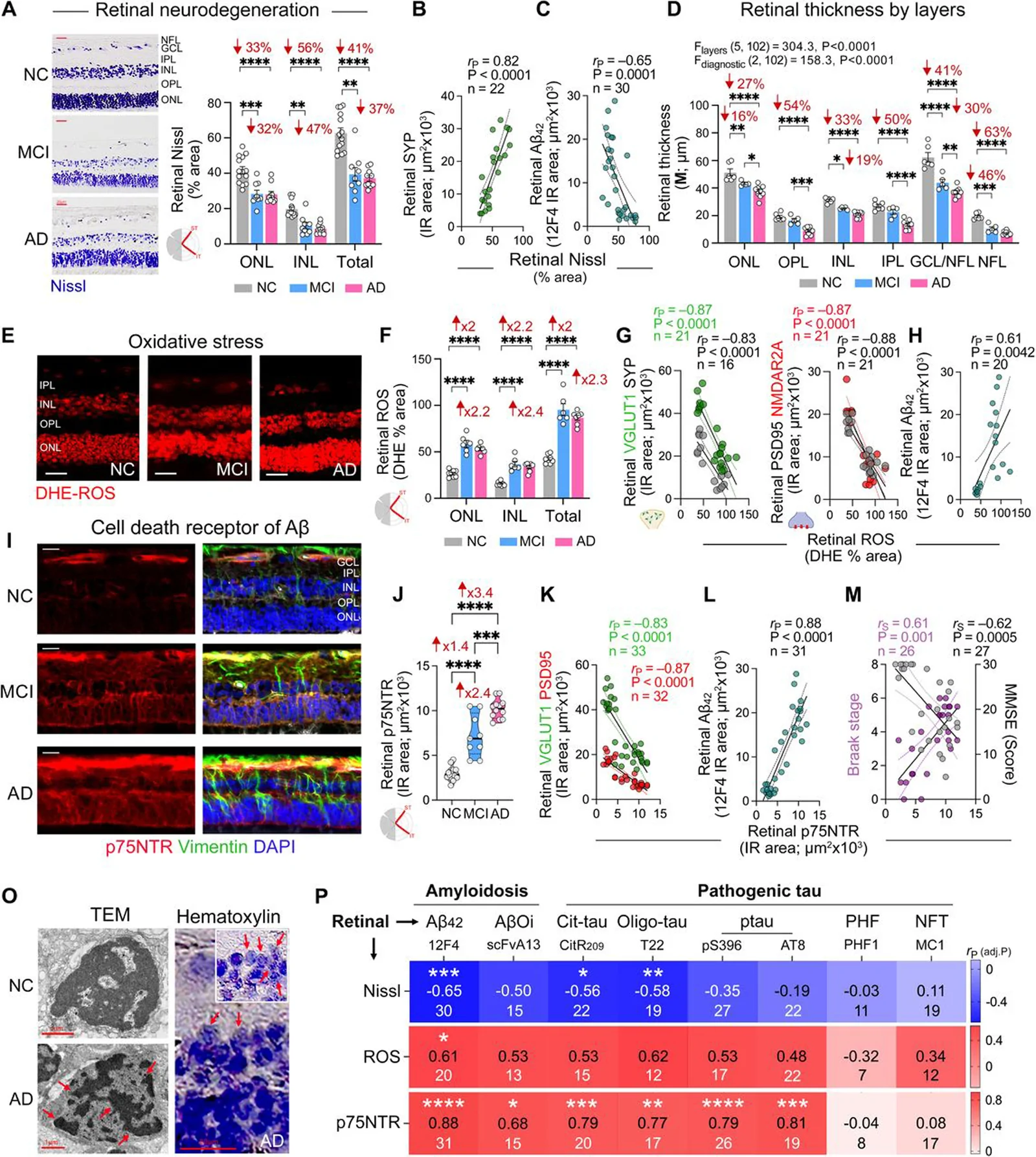
Retinal neurodegeneration and death receptor signaling in MCI and AD patient retinas. (**A**) Representative Nissl-stained retinal cross-sections from individuals with normal cognition (NC), MCI and AD dementia. Scale bars: 20 μm. Quantitative analysis of Nissl^+^ percent (%) area in the ONL, INL, and total retina (sum of GCL, INL, and ONL) of MCI (n = 9) and AD patients (n = 10) compared to NC controls (n = 14). (**B-C**) Pearson’s correlation coefficient (*r*_P_) analysis between Nissl^+^ % area and retinal (**C**) synaptophysin (SYP) and (**D**) Aβ_42_ burden. (**D**) Quantitative analysis of mid-peripheral retinal layer thickness in the superior- and inferior-temporal regions of the retina from MCI (n = 5), AD (n = 9) and NC (n = 6) individuals. (**E**) Representative images of dihydroethidium (DHE) staining showing reactive oxygen species (ROS) level in the retina from MCI, AD and NC individuals. Scale bars: 20 μm. (**F**) Quantitative analysis of retinal DHE-ROS levels in a subset of MCI (n = 6), AD patients (n = 7) and NC controls (n = 8). (**G-H**) Pearson’s correlation coefficient (*r*_P_) analysis between neuronal DHE^+^ % area and retinal (**G**) pre-synaptic markers VGLUT1 and SYP, post-synaptic markers PSD95 and NMDAR2A, and (**H**) Aβ_42_ burden. (**I**) Representative immunofluorescence images of retinal cross-sections from MCI, AD and NC individuals, labeled for p75NTR (nerve growth factor receptor; red) and vimentin (green); nuclei counterstained with DAPI (blue). Scale bars: 20 μm. (**J**) Violin plots display quantitative analysis of retinal p75NTR IR area in MCI (n=9) and AD (n=12) patients compared to matched NC controls (n = 13). (**K--L**) Pearson’s correlation coefficient (*r*_P_) analysis between retinal p75NTR versus (**K**) retinal VGLUT1 and PSD95, and (**L**) retinal Aβ_42_ burden, (**M**) Spearman’s rank correlation coefficient (*r*_S_) analysis between retinal p75NTR versus BRAAK stage and MMSE score. (**O**) TEM and H&E images showing neuronal nuclear morphology in NC and AD retinas. Shrunken, dense, and darkly stained pyknotic nuclei were observed in AD retinal cells (red arrows), indicative of cell death. (**P**) Heatmap showing Pearson’s correlations coefficient (*r*_P_) and significance (P values with Holm-Šídák multiple-comparison correction method adjusted for each retinal neuronal marker against 8 retinal synaptic, amyloidosis, and tauopathy marker) between retinal degeneration markers (Nissl, DHE-ROS, p75NTR) and retinal AD pathology, including amyloidosis (12F4^+^Aβ_42_ and scFvA13^+^ intraneuronal Aβ oligomers – AβOi) and tauopathy (pS396-tau, CitR_209_-tau, T22-tau oligomers, AT8-ptau, PHF-tau, MC1-NFTs). Violin plots show individual subjects (circles) with lower, median and upper quartiles. Bar graph displays group means ± SEMs. *P < 0.05, ** P < 0.01, *** P < 0.001, **** P < 0.0001, by one-way or two-way ANOVA followed by Tukey’s post-hoc multiple comparison test. Percentage (%) changes are shown in red. NFL, retinal nerve fiber layer; GCL, ganglion cell layer; IPL, inner plexiform layer; INL, inner nuclear layer; OPL, outer plexiform layer; ONL, outer nuclear layer.

Our protein biochemistry analyses show that synaptic protein deficits and activation of death receptor pathways have emerged as central pathological hallmarks of the AD retina. We next interrogated neurodegeneration-associated processes within anatomically defined retinal subregions and specific cellular layers in an expanded human cohort (Figure 3 and Table 3; extended data in Figure S5). To this end, we initially assessed neuronal integrity in retinas from MCI (n=9), AD (n=10), and age-matched NC controls (n=14) using neuronal-specific Nissl staining, which labels nuclei and granules (i.e., ribosomal RNA) in purple or blue (Figure 3A). Quantitative analysis revealed substantial neuronal loss across nuclear cell layers, with highly significant 32–33% losses of ONL neurons and 47–56% losses of INL neurons in MCI and AD retinas compared with NC controls (P < 0.01 – 0.001; Figure 3A). Retinal Nissl^+^ neuronal area was very strongly and directly correlated with retinal SYP (*r* = 0.82, P < 0.0001; Figure 3B) and strongly and negatively correlated with retinal Aβ_42_ burden (*r* = –0.65, P = 0.0001; Figure 3C), further supporting a link between Aβ_42_ accumulation and both neuronal and synaptic loss in the AD retina. There was also a moderate association between retinal Nissl and retinal CitR209-tau (*r* = 0.56, P = 0.05), but not with other tau isoforms (Table 3). To further characterize retinal neurodegeneration, we performed histomorphometric analysis of retinal thickness across six layers (ONL, OPL, INL, IPL, GC/NFL and NFL) in predefined C/M/F anatomical subregions (Figure 3D, mid periphery; extended data across all retinal layers and subregions are detailed in Figure S5A-I). The most noticeable thinning was detected in the mid-peripheral retina (Figure 3D), prominently in axonal- and synapse-rich layers (NFL, IPL, OPL), which were reduced by 50–63% in AD versus NC (P < 0.0001). The earliest and most pronounced loss in MCI retinas occurred in the GCL/NFL, diminished by 30–46% (P < 0.001 – 0.0001). These findings highlight region- and layer-specific vulnerability that mirrors synaptic degeneration and Aβ_42_ deposition, contributing to retinal neurodegeneration.

Pathological Aβ and tau species have been shown to drive oxidative stress^88–90^. Given the retina’s high metabolic demand, vulnerability to oxidative damage, and progressive neurodegeneration in AD, we next quantified retinal labeling related to reactive oxygen species (ROS) in the MCI and AD retina (Figure 3E-F). Dihydroethidium (DHE) labeling^91^ revealed a 2.0–2.4-fold increase in oxidized DHE fluorescence (percent area), consistent with elevated superoxide/ROS levels, in the retinal nuclear layers of MCI and AD patients compared with NC controls (P < 0.0001). Retinal DHE levels exhibited very strong inverse correlations with retinal synaptic markers (Figure 3G, Table 3), most notably PSD95 (*r* = –0.88, P_*adj.*_ < 0.0001) and NMDAR2A (*r* = –0.87, P_*adj.*_ < 0.0001), alongside strong positive correlations with 12F4^+^ Aβ_42_ area (*r* = 0.61, P = 0.0042; Figure 3H) but not with pathogenic tau species. These findings implicate oxidative stress as a downstream mediator of Aβ_42_ accumulation driving synaptic and neuronal loss in the AD retina.

Previously, p75NTR has been implicated in regulating oxidative stress, apoptosis, neuronal survival, and synaptic plasticity, and in mediating Aβ-driven neurite degeneration, tau hyperphosphorylation, and neuronal loss in AD models^72–75^, and has emerged as a top upregulated synapse-associated protein in AD retina in our MS and WB analyses (Figure 2D, 2I, 2L). Consistent with these findings, IHC analysis revealed robust increases in p75NTR expression in MCI and AD retinas (2.4–3.4-fold vs NC; *P* < 0.0001), with signal localizing predominantly to vimentin^+^ Müller glia (Figure 3I-J). Moreover, retinal p75NTR expression exhibited very strong inverse correlations with all retinal synaptic markers (Table 3), including the pre- and post-synaptic markers VGLUT1 and PSD95 (*r* = –0.83 and –0.87, respectively, P < 0.0001; Figure 3K), and a very tight direct correlation with retinal Aβ_42_ burden (*r* = 0.88, P < 0.0001, Figure 3L), supporting its role as a mediator of Aβ-induced synaptic loss in the retina, mirroring mechanisms observed in the AD brain^74,92–95^. Interestingly, strong associations between retinal p75NTR and immature but not mature tau isoforms were also observed (*r* = 0.77–0.81, P_*adj.*_ = 0.0012–0.00001, Table 3), proposing the involvement of tauopathy in p75NTR-mediated synaptic degeneration in the AD retina. We next assessed the clinical relevance of retinal p75NTR by evaluating its potential associations with brain AD pathology and cognitive status (Table 4). Retinal p75NTR expression moderate-to-strongly correlated with brain atrophy, Braak stage, and ABC scores (*r* = 0.53–0.61, P_*adj.*_ = 0.024–0.0061; Figure 3M and Table 4) and inversely associated with MMSE scores (*r* = –0.62, P = 0.0005; Figure 3M), indicating that retinal p75NTR upregulation reflects both disease severity and cognitive impairment.

TEM and hematoxylin and eosin (H&E) staining further showed the ultrastructural features of neuronal death, revealing shrunken, condensed, and darkly stained pyknotic nuclei, hallmarks of oxidative stress-induced apoptosis or necroptosis, in AD compared with NC retinas (Figure 3O, red arrows). A heatmap summary analysis (Figure 3P) highlights strong inter-relationships among retinal Aβ/tau pathologies and neurodegeneration, with retinal p75NTR emerging as a robustly correlated marker. These correlations reinforce p75NTR as a central integrator of Aβ- and tau-driven toxicity and a potential mediator of synaptic degeneration in the retina, paralleling AD mechanisms in the brain^74,92–95^.

### Retinal UCHL1 dysregulation in AD is a strong predictor of Braak stage and cognitive status.

**Figure 4.**
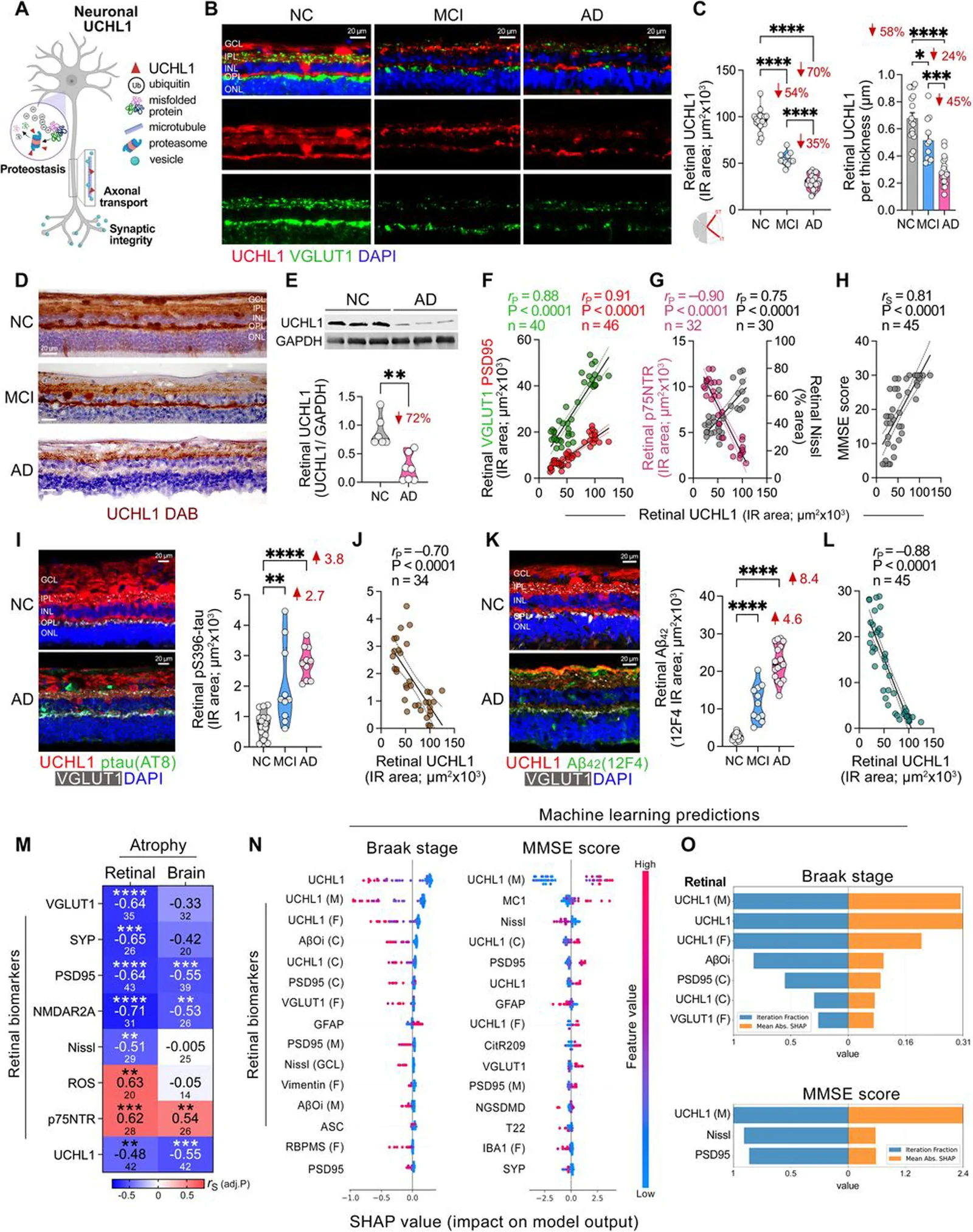
Retinal UCHL1 in relation to synapses and proteinopathies. (**A**) Schematic illustration of UCHL1 functions in neurons. (**B**) Representative fluorescent microscopic images of retinal cross sections immunolabelled for UCHL1 (red) in combination with pre-synaptic marker – VGLUT1 (green). Nuclei are labelled with DAPI (blue). Scale bars: 20 μm. (**C**) Violin plots display quantitative analysis of retinal UCHL1 IR area in patients with AD (n = 21) and MCI (n = 11) as compared with NC controls (n = 18). (**D**) Representative retinal cross-section images from MCI and AD patients compared to NC controls immunolabeled with UCHL1 (brown) using peroxidase-based DAB and hematoxylin counterstaining. Scale bars: 20 μm. (**E**) Representative Western blot showing the levels of UCHL1 in retinal lysates from AD patients versus NC (upper panel). The densitometric analyses of UCHL1, normalized to GAPDH, is shown (lower panel) (n = 7 AD, 6 NC). (**F-H**) Pearson’s or Spearman’s correlation analyses between retinal UCHL 1 IR area and the following: (**F**) retinal pre-synaptic marker - VGLUT1 (green) or post-synaptic marker - PSD95 (red), (**G**) retinal p75NTR and Nissl, and (**H**) the MMSE-cognitive scores. (**I**) Representative fluorescent images of retinal cross-sections, immunostained for UCHL1 (r ed), AT8^+^-ptau (green), and VGLUT1 (white). Nuclei are stained with DAPI (blue). Scale bars: 20 μm. The violin plot presents quantitative analysis of retinal pS396 IR area across subjects in this cohort with AD (n = 10), MCI (n= 10) and NC (n =15). (**J**) Pearson’s correlation analysis between retinal UCHL1 and retinal pS396-tau IR areas. (**K**) Representative fluorescent images of retinal cross-sections immunostained for UCHL1 (red), 12F4^+^ Aβ_42_ (green), and VGLUT1 (white). Nuclei are stained with DAPI (blue). Scale bars: 20 μm. The violin plot presents quantitative analysis of retinal 12F4^+^Aβ_42_ IR area across subjects in this cohort with AD (n = 17), MCI (n= 11) and NC (n =19). (**L**) Pearson’s correlation analysis between retinal UCHL1 IR area and retinal Aβ_42_ burden. (**M**) Heatmap showing Spearman’s rank correlation coefficient (*r*_*S*_) and significance (adjusted P values with Holm-Šídák multiple-comparison correction method for each retinal neuronal marker against multiple retinal and brain atrophy markers) between retinal (left) and brain (right) atrophy to retinal synaptic, neurodegeneration and UCHL1 markers. (**N-O**) Identification of features impacting model predictions for (N) Braak staging and (O) MMSE score. The left bars indicate the fraction of randomly-instantiated models where feature was in the top 5 features ranked by mean absolute SHAP value; the right bars indicate the mean absolute SHAP value. Violin plots show individual subjects (circles) with lower, median and upper quartiles. *P < 0.05, ** P < 0.01, *** P < 0.001, **** P < 0.0001, by unpaired Student t-test or one-way ANOVA followed by Tukey’s post-hoc multiple comparison test. Percentage (%) changes are shown in red. GCL, ganglion cell layer; IPL, inner plexiform layer; INL, inner nuclear layer; OPL, outer plexiform layer; ONL, outer nuclear layer. Schematic illustration was created using Biorender.com.

Ubiquitin carboxyl-terminal hydrolase L1 (UCHL1), a neuron-enriched deubiquitinating enzyme and key component of the UPS, is essential for proteostasis, oxidative stress responses, synaptic integrity, and neuronal survival (Figure 4A)^60,61,96^. In the brain, UCHL1 facilitates clearance of misfolded proteins and limits the aggregation of neurotoxic species such as Aβ_42_ and p-Tau^97^, whereas reduced UCHL1 expression has been linked to Aβ and pTau–mediated synaptic dysfunction and cognitive decline in AD^56,62^. We therefore evaluated UCHL1 expression and its potential association with synaptic loss and neurodegeneration in postmortem retinas from a cohort of AD (n=21, mean age 86.2 ± 8.9 years, 13 females/8 males) and MCI (n=11, mean age 89.3 ± 5.2 years, 7 females/4 males) patients, compared with NC controls (n=18, mean age 82.6 ± 10.3 years, 8 females/10 males) (Figure 4B-D; extended data in Fig. S6A-D).

In NC retinas, double immunofluorescence labeling of UCHL1 with the presynaptic marker VGLUT1 and peroxidase-based immunolabeling of UCHL1 with hematoxylin counterstaining showed intense UCHL1 expression across multiple retinal cell types, including horizontal, bipolar, amacrine, and ganglion cells. Robust expression was also evident in synapse-rich and axonal layers (OPL, IPL, NFL), whereas only sparse signal was detected in the nuclear ONL and INL (Figure 4B, 4D). Within the OPL and IPL, UCHL1 was found in close spatial proximity to VGLUT1 but exhibited limited direct co-localization, suggesting a complementary rather than overlapping synaptic distribution. In contrast, MCI and AD retinas exhibited significant decrease in UCHL1 expression in these cell types and layers, paralleling a concomitant decrease in VGLUT1 expression, indicating a tight association between UCHL1 loss and synaptic degeneration (Figure 4B). Quantitative analysis revealed significant 54–70% reductions in UCHL1 immunoreactive area in retinas from MCI and AD patients compared to NC controls (Figure 4C, left graph, P < 0.0001; Extended UCHL1 analyses of percent immunoreactive area and regional mapping, confirming 47–70% reductions across the ST/IT retina and C/M/F subregions in MCI and AD in Figure S6B-C). To determine whether reduced retinal UCHL1 simply reflects retinal thinning or neurodegeneration (significant in MCI and AD), we normalized UCHL1 to retinal thickness. The reduction persisted: UCHL1 remained significantly decreased by 24% in MCI and 45% in AD retinas (Fig. 4C, right graph, P < 0.05–0.0001). In agreement with these results, Western blot analysis in additional human cohort demonstrated a 72% decrease in UCHL1 expression normalized to GAPDH in 7 AD retinas relative to 6 NC controls (Figure 4E; a representative full WBs in Figure S6E). Proteomics showed broad UPS dysregulation in AD retinas: GSEA indicated suppression of the GO ‘protein SUMOylation’ program alongside activation of the WikiPathways ‘Parkin–ubiquitin–proteasomal system pathway’, consistent with stress-driven proteostasis and UCHL1-linked post-translational modification (Figure S7A-B). In line with prior work implicating UPS dysfunction in AD^64^, MS further revealed increased particulate/aggregated and oxidized UCHL1 and UCHL3 (Figure S7C-D), whereas our IHC and WB analyses showed reduced soluble UCHL1, together indicating a marked retinal UCHL1 imbalance in AD, paralleling reports in the AD brain^98–102^.

To assess the potential clinical significance of retinal UCHL1 in AD, we assessed its relationships with various retinal markers, brain AD-pathology, and cognitive parameters using correlation analyses (Figure 4F-H and Tables 3–4). Retinal UCHL1 exhibited robust correlations with all synaptic markers, particularly the pre-synaptic VGLUT1 and the post-synaptic PSD95 expression (*r* = 0.88–0.91, P_*adj.*_ < 0.0001; Figure 4F and Table 3). We also identified very strong to strong inverse associations between retinal UCHL1 and the cell death receptor p75NTR (*r* = –0.90, P < 0.0001), retinal neurodegeneration Nissl^+^ % area (*r* = 0.75, P < 0.0001; Figure 4G), and retinal DHE/ROS levels (*r* = –0.77, P < 0.0001; not shown). Moreover, retinal UCHL1 displayed a strong positive correlation with MMSE scores (*r* = 0.81, P_*adj.*_ < 0.0001; Figure 4H and Table 4), suggesting a direct link between retinal UCHL1 loss and cognitive decline. Notably, marked reductions in UCHL1 in MCI and AD retinas coincided with elevated pathogenic tau and Aβ species (Figure 4I–L, Table 3). With the exception of retinal mature PHF-tau and MC1^+^ tau, UCHL1 showed moderate to very strong inverse correlations with various abnormal tau and Aβ species, most prominently pS396-tau (*r* = –0.70, P_*adj.*_ < 0.0001), CitR209-tau (*r* = –0.73, P_*adj.*_ < 0.001), and Aβ_42_ burden (*r* = –0.88, P_*adj.*_ < 0.0001; Figure 4J, 4L, Table 3).

Multivariable correlations between retinal UCHL1 expression (IHC) and brain AD pathology, disease stage, and cognitive score are summarized in Table 4. Notably, UCHL1 expression inversely correlated with retinal and brain atrophy (Figure 4M), mirroring the pattern observed for synaptic markers and Nissl^+^ neuronal area. Conversely, retinal DAE-ROS levels and p75NTR expression were positively associated with retinal and brain atrophy, suggesting that oxidative stress and death receptor signaling contribute to widespread neurodegeneration observed in both the retina and brain in AD (Figure 4M). Heatmap analysis and Metascape network mapping demonstrated coordinated dysregulation of deubiquitination/UPS proteins and pathways in AD retinas (Figures S7E–F), with interconnected disruption of UPS, autophagy, axonal transport, and axonogenesis, positioning retinal UCHL1 as a central hub linking proteostasis failure to p75NTR-mediated neurodegeneration.

Finally, we trained Random Forests^103^ to predict Braak stage and MMSE score. Learning curves (Figure S7G-H) indicate that the model predicting Braak stage likely had sufficient data, but that the one for MMSE retained significant differences across folds. Both Random Forest models were trained with 80 estimators and L1 loss and used an identical retinal biomarker feature set. The mean R^2^ score across 5-fold cross-validation was 0.370 for predicting Braak stage, and 0.137 for predicting MMSE. Among all dysregulated retinal proteins, UCHL1 and PSD95 emerged as the top predictors of both Braak stage and MMSE score (Figure 4N), retinal AβOi (center) was predictive of Braak stage but not MMSE, and retinal Nissl signal and MC1 (tau tangles) were predictive of MMSE but not Braak. To mitigate sensitivity to initialization, we refit each model across 1,000 random seeds and quantified feature robustness by the consistency of their SHAP-derived importance across runs (Figure 4O). The impact of each feature on model prediction varied considerably, but UCHL1 was both consistently identified and had the greatest impact. These results highlight the potential of using non-invasive retinal synaptic biomarkers for monitoring AD progression and cognitive decline.

## Discussion

The present study reveals that retinal Aβ/p75NTR-mediated synaptic degeneration is coupled with profound dysregulation of UCHL1, which emerges as an early and defining pathological feature of AD. Across independent postmortem cohorts, including donors with MCI due to AD, we identified marked reductions in excitatory presynaptic and postsynaptic proteins, coupled with aberrant p75NTR signaling and UCHL1 imbalance, accompanied by ultrastructural collapse of synaptic architecture. These alterations were tightly associated with local Aβ_42_ and pathogenic tau species, previously reported to be elevated in AD patients^21,27–30,35–37,40,41^ and extended to measures of disease stage, brain pathology, and cognitive impairment, with retinal UCHL1 levels showing particularly strong associations. Collectively, our findings suggest that the retina undergoes a Aβ/p75NTR-mediated synaptic decline that parallels—and may faithfully reflect—the earliest cerebral synaptic pathology in AD.

A notable finding was the non-uniform spatial distribution of synaptic degeneration, with mid- and far-peripheral regions exhibiting the most pronounced deficits in MCI and AD patients compared with NC controls. The observed 50–77% reduction in pre-synaptic markers and 48–72% in post-synaptic markers highlights significant synaptic compromise even at the earliest symptomatic stages of the AD progression (MCI). This pattern of synaptic degeneration was consistent with current and prior evidence of peripheral-predominant Aβ_42_ and tau deposition in the retina^22,27,28,30,37,104^ and suggests that localized microenvironments may confer heightened vulnerability to proteinopathies. Consistent with our previous study, primary cortical neurons exhibited a 50–70% loss of glutamatergic pre- and postsynaptic densities following exposure to fibrillar or oligomeric Aβ_42_ aggregates^18^. Beyond Aβ, here we found that retinal immature and oligomeric tau isoforms, rather than mature PHF- or MC1-positive tau aggregates, were tightly correlated with synaptic loss, echoing accumulating evidence from the brain that soluble, non-fibrillar tau species exert disproportionate synaptotoxic effects^105–107^.

Retinal proteome analysis highlighted a dual vulnerability in the AD retina: retinal synaptic failure and photoreceptor degeneration. Photoreceptor degeneration was determined by broad downregulation of photoreceptor proteins, including opsins and other critical elements of the phototransduction cascade. Analysis of synapse-associated proteins indicated dysregulation of critical molecular pathways related to the synaptic vesicle cycle, neuronal synaptic plasticity, and dendritic spine development. Decreased levels of positive regulators of synaptic docking—including SYP, UNC13A, and STXBP1—and increase of negative regulators of synaptic endocytosis—including BIN1, ARF6, and CLTB—indicate the disruption of neurotransmitter release and synaptic vesicle recycling, respectively^108–114^. Other key positive regulators of synaptic transmission—such as ADCY1, EPHB2, GNB5 and PSD95^111,115,116^—were largely downregulated. Together with GSEA suggesting dysregulated synaptic related pathways, these alterations suggest impaired vesicle cycling and reduced excitatory transmission, converging on mechanisms of functional disconnection previously established in hippocampal and cortical circuits^117–121^.

The robust upregulation of p75NTR emerged as a particularly striking feature of the AD retina. A death receptor, p75NTR plays key roles in axon pruning, synaptic plasticity, and apoptosis in the CNS ^73,122^. In AD models, Aβ binding to p75NTR induces neuritic degeneration, tau hyperphosphorylation, oxidative stress, and neuronal loss^72,74,75^. Elevated p75NTR levels correlated closely with reduced synaptic proteins (VGlut1, PSD95), increased Aβ_42_ burden, immature tau isoforms, and elevated oxidative stress, suggesting its role as a mediator of convergent neurotoxic pathways. Given the ability of Aβ to bind p75NTR and trigger apoptotic signaling^76,86,87^, our data support the possibility that p75NTR integrates multiple pathogenic inputs, Aβ, tau, and oxidative stress, culminating in synaptic degeneration. These findings align with prior work in AD models demonstrating that p75NTR contributes to neuritic degeneration, tau hyperphosphorylation, and neuronal loss in the AD retina^74,75,123^.

A central and novel observation is the dysregulation of UCHL1, a neuron-enriched deubiquitinating enzyme critical for proteostasis and synaptic integrity^61,124^. UCHL1 expression was markedly reduced in MCI and AD retinas, particularly in synapse-rich layers, while mass spectrometry revealed accumulation of particulate and oxidized forms. UCHL1 is susceptible to ROS and oxidized UCHL1, which is prone to aggregation, accumulates in AD brains^98,99^, whereas soluble UCHL1 levels and activity are reduced^101^. The antibody used in this study primarily recognizes the cytosolic, soluble form of UCHL1, which has been reported to be reduced in brain lysates from patients with AD and Parkinson’s disease^60,125^. This reduction may explain the diminished UCHL1 signal observed in the AD retina through fluorescence and non-fluorescence histological analyses and further validation by biochemical analysis should demonstrate the soluble form of UCHL1. In contrast, mass spectrometry likely detects the oxidized form. This imbalance echoes brain studies linking UCHL1 dysfunction to impaired clearance of Aβ and tau aggregates^126,127^. In our cohorts, retinal UCHL1 strongly correlated with synaptic markers, p75NTR, ROS levels, pathogenic tau isoforms, Aβ_42_ burden, and cognitive performance. Network analyses further implicated retinal UCHL1 in interlinked processes of autophagy, axonal transport, and synaptic recycling, suggesting that loss of soluble UCHL1 may represent a nodal point at which multiple pathogenic cascades converge. Indeed, our data suggests that UCHL1 sits at the crossroads of different disease mechanisms (proteostasis, oxidative stress, tau and Aβ toxicity, synaptic dysfunction) and its dysregulation could connect these otherwise separate processes into a common retinal and brain neurodegenerative pathway.

Correlative analyses of both retinal and brain pathologies revealed that both synaptic markers and UCHL1 track with Braak stages, brain atrophy, and cognitive impairment. Importantly, AI-based multivariable modeling identified retinal UCHL1 as the top predictor of disease stage and MMSE score, underscoring its translational potential as a non-invasive biomarker. While these findings cannot establish causality, they highlight the retina as a uniquely accessible site in which to interrogate synaptic and proteostatic dysfunction in AD.

Several limitations warrant consideration. Postmortem analyses cannot capture longitudinal trajectories or establish causal mechanisms, and differences in tissue preservation may affect protein integrity. Cohort sizes, while substantial for retinal neuropathological studies, remain modest for multivariable modeling. Finally, although retinal findings closely parallel those observed in the brain, the extent to which retinal pathology reflects systemic versus locally driven processes remains unresolved. Future work integrating in vivo imaging of retinal UCHL1 or synaptic integrity with functional and mechanistically targeted studies will be essential to validate these findings and assess their diagnostic utility.

In summary, our results position the retina as a critical site of early synaptic degeneration, proteostatic imbalance, and death receptor signaling in AD. The convergence of Aβ_42_, tau, oxidative stress, p75NTR, and UCHL1 dysregulation highlights interconnected mechanisms that likely contribute to both retinal and cerebral neurodegeneration. By revealing retinal UCHL1 as a strong predictor of disease stage and cognitive impairment, these findings provide a foundation for the development of novel retinal biomarkers. Such advances may deepen our understanding of AD pathophysiology and provide new means for clinically monitoring the progression of the disease against various conditions including purported new treatments.

### Lead contact

Further information and requests for resources and reagents should be directed to and will be fulfilled by the lead contact, Maya Koronyo-Hamaoui (maya.koronyo@csmc.edu)

### Material availability

This study did not generate new unique reagents.

### Data and code availability

Mass Spectrometry data are publicly available via the PRIDE database under the ProteomeXchange accession #PXD040225. All data are available in the main text, tables, and figures or supplementary materials (Figure S1-S7 and Tables S1-S7). All other material or data requests should be addressed to the corresponding author. The Jupyter notebook containing the analysis with random forests has been uploaded to https://github.com/xomicsdatascience/ubiquitin_ad. Data will become publicly available as of the date of publication.

## STAR METHODS

### EXPERIMENTAL MODEL AND STUDY PARTICIPANT DETAILS

#### Postmortem Human eye and brain samples

Postmortem human eye globes and brain tissues were obtained from the Alzheimer’s Disease Research Center (ADRC) Neuropathology Core in the Department of Pathology at the University of Southern California (USC, Los Angeles, CA; IRB protocol HS-042071). In addition, eye globes were obtained from the National Disease Research Interchange (NDRI, Philadelphia, PA; IRB exempt protocol EX-1055). For a subset of patients and controls, brain specimens were also obtained from the ADRC Neuropathology Core at the University of California, Irvine (UCI [IRB protocol HS#2014–1526]). USC-ADRC, NDRI, and UCI ADRC maintain human tissue collection protocols that are approved by their respective governing committees and are subject to oversight by the National Institutes of Health and institutional guidelines. All the histological procedures were conducted at Cedars-Sinai Medical Center under IRB protocols (Pro00053412 and Pro00019393, Pro00055802). For histological examinations, 53 retinas were collected from deceased donors with confirmed AD (n = 21) or MCI due to AD (n = 11), and from age- and sex-matched deceased donors with NC (n = 21). For analysis of retinal proteins using mass spectrometry (MS), Enzyme-Linked Immunosorbent Assay (ELISA) and Western blot (WB), fresh-frozen retinas were collected from another cohort of deceased donors (n = 16) with clinically and neuropathologically confirmed AD patients (n = 9) and matched NC controls (n = 7). Comprehensive cohort details are presented in Table 1, and Table S1. The human cohort used in this study exhibited no significant differences in age, sex, or post-mortem interval (PMI) hours. Patients' confidentiality was maintained by de-identifying all tissue samples, ensuring that donors could not be identified.

#### Clinical and neuropathological assessments

The detailed clinical and neuropathological assessment procedures are described in our recent publication^28,37^. In summary, clinical and neuropathological reports detailing patients' neurological examinations and neuropsychological and cognitive assessments were generously provided by the ADRC system using through the Unified Data Set^129^. The NDRI provided patient information, including sex, ethnicity, age at death, cause of death, medical history indicating AD, the presence or absence of dementia, and any accompanying medical conditions. Most cognitive assessments were conducted annually, typically within one year prior to death. In this study, we utilized cognitive scores obtained closest to the patient’s time of death. Three global indicators of cognitive status were used for clinical assessment: the Clinical Dementia Rating (CDR scores: 0 = normal; 0.5 = very mild impairment; 1 = mild dementia; 2 = moderate dementia; or 3 = severe dementia)^130^, Montreal Cognitive Assessment (MOCA scores: ≥26 = cognitively normal or <26 = cognitively impaired^131,132^, and the Mini-Mental State Examination (MMSE scores: normal cognition = 24 – 30; MCI = 20 – 23; moderate dementia = 10 – 19; or severe dementia ≤ 9)^133^.

The assessment of cerebral Aβ burden comprised analysis of diffuse and neuritic plaques (including both immature and mature forms), along with amyloid angiopathy, neurofibrillary tangles (NFTs), neuritic threads (NTs), granulovacuolar degeneration, Lewy bodies, Hirano bodies, Pick bodies, ballooned neurons, neuronal loss, microvascular changes, and gliosis. These evaluations were conducted across multiple brain regions, notably in the hippocampus, the entorhinal cortex, the superior frontal gyrus in the frontal lobe, the superior temporal gyrus in the temporal lobe, the superior parietal lobule in the parietal lobe, the primary visual cortex, and the visual association area in the occipital lobe. All brain samples were uniformly collected by a neuropathologist.

Formalin-fixed, paraffin-embedded brain sections were used to determine the severity of amyloid plaques and NFTs in the brain using anti Aβ monoclonal antibody (mAb) clone 4G8, anti phospho-tau mAb clone AT8, Thioflavin-S (ThioS), and Gallyas silver staining. Two neuropathologists independently rated the burden of Aβ, NFTs, and NTs on a scale of 0, 1, 3, and 5 [(0 = none, 1 = sparse (0–5), 3 = moderate (6–20), 5 = abundant/frequent (21–30 or above), N/A = not applicable)], with final scores calculated as the average of the two readings. The final diagnosis included AD neuropathologic change. The Aβ plaque scoring system, was adapted from Tal et al., (A0 = no Aβ or amyloid plaques, A1 = Thal phase 1 or 2, A2 = Thal phase 3, and A3 = Thal phase 4 or 5)^134^. NFT staging was adjusted from Braak for silver-based histochemistry or p-tau immunohistochemistry (B0 = No NFTs, B1 = Braak stage I or II, B2 = Braak stage III or IV, B3 = Braak stage V or VI)^135^. The neuritic plaque score was adapted from CERAD (C0 = no neuritic plaques, C1 = CERAD sparse, C2 = CERAD moderate, C3 = CERAD frequent)^136^. Additional evaluations included neuronal loss, gliosis, granulovacuolar degeneration, Hirano bodies, Lewy bodies, Pick bodies, and ballooned neurons, using hematoxylin and eosin staining, with scores of 0 for absent and 1 for present. Amyloid angiopathy was classified into 4 grades: Grade I indicates amyloid around normal/atrophic smooth muscle cells of vessels; Grade II shows media replaced by amyloid without blood leakage; Grade III involves extensive amyloid deposition with vessel wall fragmentation and perivascular leakage; Grade IV includes extensive amyloid deposition with fibrinoid necrosis, microaneurysms, mural thrombi, lumen inflammation, and perivascular neuritis.

### METHOD DETAILS

#### Collection and processing of eyes tissues

Donor eyes were obtained within an average of 7.9 hours from the time of death. For histological and TEM analyses, eyes were punctured once at the limbus, fixed in either 10% neutral buffered formalin or 4% paraformaldehyde (PFA), and stored at 4°C. Freshly collected eyes for biochemical analyses were preserved in Optisol-GS media (Bausch & Lomb, 50006-OPT) and stored at 4°C for less than 36 hours, after which they were snap frozen and stored at −80°C. These procedures were uniformly performed across all tissue sources and collection sites.

#### Preparation of retinal tissues and cross-sections

Eyes that were either freshly preserved in Optisol-GS or fixed were dissected on ice. After removal of the anterior chamber to obtain eyecups, the vitreous humor was carefully cleared. Retinas were then meticulously isolated, separated from the choroid, and prepared as flatmounts following established procedures^27,28,37^. Flatmount strips (~2mm wide) extending from the ora serrata to the optic disc were dissected to generate four strips: superior-temporal (ST), inferior-temporal (IT), inferior-nasal (IN), and superior-nasal (SN). The ST and IT strips analyzed in this study, generated from fixed retinas, were embedded in paraffin, rotated 90° horizontally, and re-embedded in paraffin blocks. These retinal strips, encompassing central, mid, and far retinal subregions, were sectioned at 8–10 μm and mounted on microscope slides treated with 3-Aminopropyltriethoxysilane (APES, Sigma A3648). Temporal hemiretinas dissected from fresh tissue was stored at − 80 °C for downstream protein analysis (MS, ELISA, WB). Together, these preparation methods provided consistent and comprehensive access to retinal quadrants, layers, and pathological subregions.

#### Immunohistochemical analysis (IHC)

Before the IHC procedure, paraffin-embedded retinal cross-section slides were deparaffinized in 100% xylene twice (10 min each), rehydrated through decreasing concentrations of ethanol (100% to 70%), and washed with distilled water followed by PBS. Antigen retrieval was performed by incubating retinal cross-sections in target retrieval solution (pH 6.1; S1699, DAKO) at 99 °C for 1 h, followed by PBS washes. In some staining cases, sections were additionally treated with 70% formic acid (ACROS) for 10 min at room temperature (RT).

##### Peroxidase-based IHC:

labelling was performed using the Vectastain Elite ABC HRP kit (Vector, PK-6101, Peroxidase rabbit IgG) according to the manufacturer’s instructions. In summary, after incubation with 3% H_2_O_2_ for 20 min, sections were washed in PBS and incubated with blocking serum containing 0.25% Triton X-100 (Sigma, T8787) for 45 min at RT. Primary antibodies (key resources table) were diluted in PBS containing blocking serum and applied overnight at 4 °C. The following day, sections were washed three times in PBS, incubated with secondary antibody for 30 min at 37°C, washed again three times in PBS and incubated with ABC reagent for 30 min at RT. After PBS washes, signal was visualized with 3,3′-diaminobenzidine (DAB) substrate (DAKO K3468). Hematoxylin counterstaining was performed, and slides were mounted with Paramount aqueous mounting medium (DAKO, S3025). Control sections were processed without the primary antibodies to assess nonspecific labeling.

##### Fluorescence-based immunostaining:

sections were treated with blocking solution (DAKO X0909) containing 0.25% Triton X-100 (Sigma, T8787) for 1 h at RT, followed by overnight incubation at 4°C with primary antibodies diluted in the same blocking solution. The following day, after PBS washes, sections were incubated with fluorophore-conjugated secondary antibodies (key resources table) for 1 hour at RT. After PBS washes, sections were mounted using Fluoromount-G, with DAPI (Invitrogen, #00–4959-52). Control sections processed without the primary antibodies were used to assess nonspecific fluorescence.

#### Nissl staining

Neuronal cytoplasm was specifically stained with the Nissl staining technique. Deparaffinized and rehydrated sections were stained in 0.1% Cresyl Violet acetate (Sigma #C5042) for 5 min, rapidly rinsed in tap water, and briefly dipped in 70% ethanol. Sections were then dehydrated through 2 changes of absolute ethanol for 3 min each, followed by immersion in xylene twice for 2 min, and mounted using mounting medium xylene (Fisher scientific company, L.L.C. #245–691). An average of 12 images per section, covering the retinal neurons from the optic disc to the ora serrata, were captured at a 20x objective and analyzed to quantify the percent neuronal area in the ONL and INL.

#### Dihydroethidium (DHE) labeling

Tissues were fluorescently labeled with DHE (Sigma-Aldrich #D7008) for 30 min at 37°C (10 μM final concentration in PBS) prior to mounting.

#### Microscopy and Quantitative immunohistochemistry

Fluorescence and bright field images were acquired using a Carl Zeiss Axio Imager Z1 fluorescence microscope with ZEN 2.6 blue edition software (Carl Zeiss MicroImaging, Inc.) and equipped with ApoTome, AxioCam MRm, and AxioCam HRc cameras. Multi-channel image acquisition was used to create images with several channels. Images were repeatedly captured at 20× (resolution of 1388 × 1040 pixels, 447.63 μm × 335.30 μm /per image) or 40× (resolution of 1388 × 1040 pixels, 223.82 μm × 167.70 μm /per image) at the same focal planes with the same exposure time for each marker and human donor. We randomly acquired 3 images from the central, 4 from the mid-, and 3 from the far-retinal subregions, for analytical purposes. Images were exported to Fiji ImageJ (version 2.14.0) to analyze parameters of interest. Acquired images were converted to grayscale and standardized to baseline by using a histogram-based threshold in Fiji ImageJ. This baseline-derived threshold was then applied uniformly to the corresponding single channel for all subjects across diagnostic groups. Images were subsequently subjected to ImageJ2/Fiji particle analysis for each biomarker to determine total and % immunoreactive area. Thickness measurements in μm (from the inner limiting membrane to the outer limiting membrane) were manually assessed using ZEN 2.6 Blue Edition software. Throughout the analysis process, the researchers were blinded to each patient’s diagnosis.

#### Transmission Electron Microscopy (TEM)

Tissue samples were initially fixed in 4% paraformaldehyde (PFA) in phosphate-buffered saline (PBS) for 20 min at room temperature (RT), followed by three washes in PBS (10 min each). For intracellular antigen labeling, samples were permeabilized and blocked in PBS containing 1% bovine serum albumin (BSA) and 0.1% Triton X-100 for 15 min at RT, then rinsed in PBS. Primary antibodies against PSD95 and VGLUT1 were diluted in blocking solution (1% BSA in PBS) and applied overnight at 4°C in a humidified chamber. Samples were then washed three times with PBS (5 min each). Secondary detection was performed using species-specific, gold-conjugated secondary antibodies (anti-rabbit, 6 nm; anti–guinea pig, 12 nm), diluted in PBS containing 1% BSA. Samples were incubated for 1 hour at RT, followed by three additional PBS washes (5 min each). Subsequently, samples were post-fixed in half-strength Karnovsky’s fixative (2% paraformaldehyde, 2.5% glutaraldehyde in 0.1 M cacodylate buffer, pH 7.2) for 15 min. Following post-fixation, the samples were rinsed in 0.1 M cacodylate buffer (pH 7.2), then post-fixed in 2% osmium tetroxide for 1 hour, rinsed again in cacodylate buffer, and subsequently in sodium acetate. En bloc staining was performed using 1% aqueous uranyl acetate for 1 hour, followed by a wash in sodium acetate. Samples were then dehydrated through a graded series of ethanol (50%, 70%, 85%, 95%, and 100%), followed by incubation in 1:1 and 1:2 mixtures of ethanol and propylene oxide (PO), and finally in pure PO. Tissues were infiltrated with PO/Eponate resin mixtures and embedded in 100% Eponate resin. Polymerization was carried out at 60°C for 48 hours. Ultrathin sections (60 nm) were obtained using an ultramicrotome and collected on copper grids. Sections were imaged using a JEOL JEM-2100 LaB6 transmission electron microscope operating at 120 kV (JEOL USA). Digital images were captured with an Orius SC1000B CCD camera (Gatan).

#### Proteome analysis by mass spectrometry (MS)

In this study, we performed secondary analyses on previously published retinal mass spectrometry data ^28^, to identify DEPs associated with synaptic function and integrity. In brief, this analysis included the following steps: (1) preparation of retinal and brain homogenates; (2) tandem mass tag labeling; (3) nanoflow liquid chromatography electrospray ionization tandem mass spectrometry; and (4) database searching, peptide quantification, and statistical analysis. Given the exploratory nature and limited sample size of this human retinal dataset, we adopted a less stringent threshold, defining DEPs as proteins with an FDR-adjusted P < 0.20 and |FC| > 1.2 (or, in selected sub-analyses, an unadjusted P < 0.05 and |FC| > 1.2, as specified for each analysis) for downstream interpretation.

##### Functional Network and Computational Analysis:

prediction of activation or inhibition of pathways related to retinal degeneration, synaptic function and integrity, and neurotransmission were analyzed with Ingenuity Pathway Analysis (IPA, Qiagen; https://digitalinsights.qiagen.com). Activation z-scores, P-values and Benjamini-Hochberg adjusted P-values are reported. Volcano plots representing expression changes [log_2_(FC)] and significance levels [-log_10_(P)] in AD versus NC was created using Prism 10.3.1 (GraphPad) and included human protein related to synapse. The list of synaptic-associated proteins was extracted from SunGO release 1.2 knowledge base. Gene Ontology (GO) analysis of synapse-related differentially expressed proteins (DEPs) was performed in Metascape (https://metascape.org/; cutoffs: overlap ≥ 3, *P*-value < 0.01, enrichment ≥ 1.5) and included the GO Biological Processes, Reactome, Kyoto Encyclopedia of Genes and Genomes (KEGG) and WikiPathways databases. Pathway networks were created in Metascape and subsequently loaded and modified in Cytoscape 3.10.2 (https://cytoscape.org/). Protein interaction networks were generated in String v12.0 and modified in Cytoscape. Heatmaps corresponding to the protein expression level of select proteins in the retina of the 6 NC individuals and the 6 AD patients, standardized by unit variance scaling, were generated in ClustVis (https://biit.cs.ut.ee/clustvis/). The mass spectrometry proteomics data have been deposited to the ProteomeXchange Consortium via the PRIDE^137^ partner repository under the dataset identifier PXD040225 (https://www.ebi.ac.uk/pride/archive/projects/PXD040225; https://ftp.pride.ebi.ac.uk/pride/data/archive/2025/10/PXD040225).

##### Generation of Gene-level Ranking Scores and Gene Set Enrichment Analysis:

Differential protein abundance statistics were processed to generate a continuous, directionally signed ranking metric suitable for preranked gene set enrichment analysis (GSEA)^85^. For each quantified protein, the log fold-change direction (positive or negative) and the raw P-value from the comparison of interest were used to compute a score defined as: rank_score=sign(logFC)×[−log10(P)]. This metric integrates the magnitude and direction of differential abundance with the statistical strength of the evidence, producing a continuous distribution of values that preserves ordering across proteins. Proteins lacking a gene symbol or P-value were removed. When multiple entries mapped to the same gene symbol, scores were averaged to produce a single representative value per gene. The resulting gene-level scores were sorted in descending order to generate the ranked list required for preranked GSEA.

Pathway enrichment analysis was performed using the *gseapy* Python package (https://github.com/zqfang/gseapy), which implements the Broad Institute’s GSEA algorithm. The prerank module was run with 1,000 permutations, a minimum gene set size of 15, and a maximum gene set size of 500. Gene sets were obtained from the designated human pathway collections (e.g., KEGG_2021_Human, GO Biological Process, or WikiPathways, depending on the analysis). Normalized enrichment scores (NES), nominal P-values, and false discovery rate (FDR) values were extracted from the gseapy output. Selected pathways are shown. Enrichment plots and ranked-metric distributions were generated directly using gseapy’s plotting utilities.

#### Biochemical determination of Aβ_1–42_ levels by sandwich ELISA in human retina

In this study, we reused retinal Aβ_1–42_ data generated in our previous study^28^ to perform pairwise correlation analyses with newly identified differentially expressed proteins. Fresh-frozen human retinal strips from the temporal hemisphere were homogenized as previously described^27,28^. The amount of Aβ_1–42_ in retinal homogenates was determined using an anti-human Aβ_1–42_ end-specific sandwich ELISA kit (Thermo Fisher, KHB3441) and normalized to the total protein concentration (Thermo Fisher Scientific).

##### Western blot analysis of human retina:

Fresh-frozen postmortem temporal hemiretina tissues from human donors were homogenized in radioimmunoprecipitation assay (RIPA) buffer (0.5M Tris-HCl, pH 7.4, 1.5M NaCl, 2.5% deoxycholic acid, 10% NP-40, 10mM EDTA, Millipore; 20–188) supplemented with protease and phosphatase inhibitors. Protein concentration was determined using a bicinchoninic acid protein assay kit (Pierce^™^). Lysates were cleared with brief centrifugation for 10 min at 8000g, normalized, and boiled at 95°C after addition of 6X SDS loading dye. Equal amounts of protein (30 μg per sample) were separated on 4–20% precast polyacrylamide gels (Bio-Rad, catalog #4561094) and transferred to polyvinylidene difluoride membranes. The membranes were then blocked with 2.5% bovine serum albumin in 1x TBS-T (Tris-buffered saline with 0.1% Tween-20) for 1 h at RT, followed by overnight incubation at 4 °C with the anti-UCHL1, anti-PSD95, anti-synaptophysin, and anti-p75NTR primary antibody (key resources table). After washing with 1x TBS-T, the membrane was incubated with fluorescently labeled secondary antibodies, and bands were detected using the LI-COR Odyssey imaging system. Band intensities were quantified using Image Studio software (LI-COR), and relative protein expression levels were calculated by normalizing target protein signals to GAPDH. In some cases, membranes were re-probed with primary antibodies after stripping with 1X stripping buffer (NewBlot^™^ Nitro stripping buffer, Licorbio #928–40030).

##### Machine learning prediction:

The dataset was split into training (80%, 38/14/44 NC/MCI/AD) and test sets (20%, 10/4/10 NC/MCI/AD), stratified by clinical diagnosis and patient sex. Two random forest regression models were trained to predict Braak stage and MMSE using 80 estimators and L1 loss, with both models using the same set of retinal biomarkers for their predictions. Due to random forest models being sensitive to their initial conditions, we instantiated the models using 1,000 different random seeds to identify which features were robustly predictive of their target based on their SHAP values^138^. At each iteration, the top features sorted by mean absolute SHAP were tabulated. After all iterations, these features were gathered and the fraction of iterations where they were present was computed (1.0 signifies the feature was in all iterations; 0.5 in half). Learning curves were produced using 10 steps between 10% and 100% of data; cross-validation scores were obtained using 5-fold cross-validation and taking the mean across folds.

### QUANTIFICATION AND STATISTICAL ANALYSIS

All statistical analyses were conducted using GraphPad Prism 10.0.3 (GraphPad Software). Data are presented as mean ± standard error of the mean (SEM) for bar graphs, or as lower quartile, median, and upper quartile values for violin plots. Comparisons among three or more groups were performed using one-way or two-way analysis of variance (ANOVA), followed by Tukey’s post hoc test for multiple comparisons. Comparisons between two independent groups were assessed using two-tailed unpaired Student’s t-tests. Associations between continuous variables were evaluated using Pearson’s correlation coefficient (*r*) for normally distributed data or Spearman’s rank correlation coefficient (*r*) for non-parametric data. Pairwise correlation *r or r* and P values were reported to indicate the direction, strength, and significance of the linear relationship. For multivariable correlation analyses, P values were further adjusted for multiple comparisons using the Holm–Šídák method, and P_*adj.*_ are reported. A P value of <0.05 was considered statistically significant. Where applicable, fold changes (FC) and corresponding 95% confidence intervals (CI) were calculated to quantify the magnitude and precision of observed effects.

## Supplementary Material

Supplement 1

Supplemental information

Figures S1-S7

Tables S1-S7

## Figures and Tables

**Table 1.** Demographic and neuropathological data on human brains and retinas in this study.

Histology cohort		NC	MCI	AD	*F*	P

N = 53 [F (%), M]		21 [11F (52%), 10M]	11 [7F (64%), 4M]	21 [13F (62%), 8M]	–	–
Age at death (years)		82.6 ± 10.5	89.3 ± 5.2	86.2 ± 8.9	2.11	0.13
Race		16W (76%), 2B, 3H	9W (82%), 1B, 1H	15W (71%), 4A, 2H	–	–
PMI (hours)		7.8 ± 4.0	8.2 ± 4.9	7.8 ± 3.6	0.05	0.95
MMSE score (N =46)		28.8 ± 2.0	21 ± 7.2	12.5 ± 7.6	33.0	**<0.0001**
CDR score (N=36)		0.2 ± 0.4	2.1 ± 1.2	2.1 ± 0.9	15.8	**<0.0001**
**Brain neuropathology**	Braak stage (%)	0-II 73%	0-II 27%	0-II 0%		
(N =43)		III-IV 18%	III-IV 45%	III-IV 24%	24.4	**<0.0001**
		V-VI 9%	V-VI 27%	V-VI 76%		
	ABC average	1.4 ± 0.8	2.1 ± 0.5	2.8 ± 0.3	24.6	**<0.0001**
	Aβ plaque^#^	1.1 ± 1.1	1.90 ± 0.8	2.6 ± 1.0	9.2	**0.0005**
	NFTs^#^	0.5 ± 0.5	1.6 ± 1.0	2.3 ± 1.1	13.5	**<0.0001**
	NTs^#^	0.4 ± 0.8	1.1 ± 0.8	1.2 ± 0.8	3.7	**0.036**
	Atrophy^#^	0.5 ± 0.7	1.1 ± 0.9	1.7 ± 1.1	5.7	**0.0067**
Protein biochemistry cohort		NC		AD		P

N = 16 [F (%), M]		7 [4F (57%), 3M]		9 [6F (67%), 3M]		–
Age at death (years)		76.7 ± 5.4		82.0 ± 15.7		0.41
Race		6W (86%), 1H		8W (89%), 1H		–
PMI (hours)		7.5 ± 4.2		6.6 ± 2.6		0.67
MMSE score (N =4)		23.0 ± n.a.		10.7 ± 6.1		0.22
CDR score (N=7)		0.3 ± 0.4		2.4 ± 0.9		**0.025**
**Brain neuropathology**	Braak stage (%)	I-II (100%)		III (14%)		**0.0013**
(N=9)		–		V-VI (86%)		
	ABC average	1.7 ± 0.5		2.6 ± 0.5		**0.044**
	Aβ plaque^#^	1.7 ± 0.7		2.8 ± 1.0		0.25
	NFTs^#^	1.2 ± 0.007		2.7 ± 1.1		0.088
	NTs^#^	0.2 ± 0.1		1.3 ± 0.7		0.074
	Atrophy^#^	0.6 ± 0.07		1.2 ± 1.0		0.42

List of human donors (N = 69 subjects) included in this study. Paired brains with neuropathological assessments were available for 52 human donors.

Mean ABC scores were determined as follows: A, Aβ plaque score modified from Thal; B, NFT stage modified from Braak; C, neuritic plaque score modified from CERAD.

Group values are presented as mean ± standard deviation. *F* and P-values were determined using one-way analysis of variance with Tukey's multiple comparisons test. P-values presented in bold type demonstrate significance.

# Severity score.

*Abbreviations*: Aβ, amyloid beta; AD, Alzheimer's disease; A, Asian; B, Black; CDR, Clinical Dementia Rating; F, female; H, Hispanic; M, male; MCI, mild cognitive impairment; MMSE, Mini-Mental State Examination; NC, cognitively normal controls; NFTs, neurofibrillary tangles; NTs, neuropil threads; PMI, postmortem interval; W, White.

**Table 2.** Correlations between retinal synaptic markers and retinal AD pathology.

Retinal	Amyloidosis		Tau isoforms					
	Aβ_42_	AβOi	CitR209	T22	pS396	AT8	PHF	MC1
**VGLUT1**	−0.81**** 38	−0.66** 19	−0.64** 25	−0.72** 21	−0.72**** 32	−0.48 22	−0.074 10	−0.18 21
**SYP**	−0.87**** 26	−0.83** 13	−0.73** 21	−0.65* 16	−0.81**** 21	−0.60* 18	−0.58 8	−0.19 16
**PSD95**	−0.86**** 44	−0.50* 25	−0.68** 25	−0.68** 21	−0.70**** 33	−0.68** 23	0.018 11	−0.021 21
**NMDAR2A**	−0.79**** 32	−0.59* 18	−0.74*** 23	−0.62* 20	−0.72*** 28	−0.49 20	−0.069 10	−0.14 20

Pearson’s correlation analyses showing the strength of the association (*r*, upper value), sample size number (n, lower value), and adjusted P values with Holm-Šídák multiple-comparison correction method (adjusted for each retinal synaptic marker against 8 retinal amyloidosis and tauopathy markers):

* P < 0.05,

** P < 0.01,

*** P < 0.001,

**** P < 0.0001.

**Table 3.** Correlations between retinal neurodegeneration-related and UCHL1 markers with retinal AD pathology.

Retinal	Synaptic markers				Amyloidosis		Tau isoforms					
	VGLUT1	SYP	PSD95	NMDAR2A	Aβ_42_	AβOi	CitR209	T22	pS396	AT8	PHF	MC1
**Nissl**	0.68*** 31	0.82**** 22	0.69*** 32	0.79**** 26	−0.65*** 30	−0.50 15	−0.56* 22	−0.58 19	−0.35 27	−0.19 22	−0.03 11	0.11 19
**ROS (DHE)**	−0.87**** 21	−0.83*** 16	−0.88**** 21	−0.87**** 21	0.61* 20	0.53 13	0.53 15	0.62 12	0.53 17	0.48 12	−0.32 7	0.34 12
**p75NTR**	−0.83**** 33	−0.84**** 22	−0.87**** 32	0.81**** 27	0.88**** 31	0.68* 15	0.79*** 20	0.77** 17	0.79**** 26	0.81*** 19	−0.039 8	0.076 17
**UCHL1**	0.88**** 40	0.89**** 27	0.91**** 46	0.86**** 34	−0.88**** 45	−0.57** 25	−0.73*** 25	−0.65** 23	−0.70**** 34	−0.63** 22	−0.21 10	−0.13 23

Pearson’s correlation analyses showing the strength of the association (*r,* upper value), sample size number (n, lower value), and adjusted P values with Holm-Šídák multiple-comparison correction method (adjusted for each retinal neuronal marker against 12 retinal synaptic, amyloidosis, and tauopathy markers):

* P < 0.05,

** P < 0.01,

*** P < 0.001,

**** P < 0.0001.

**Table 4.** Correlations between retinal UCHL1 and neurodegeneration-related markers with brain pathology and cognition

Brain	Aβ plaques	NFTs	Atrophy	Braak	ABC	CDR	MMSE
**Retina Nissl**	−0.39 25	−0.44 25	−0.005 25	−0.30 25	−0.35 25	−0.64** 25	0.64** 27
**ROS (DHE)**	−0.081 14	−0.077 14	−0.046 14	−0.13 14	0.28 14	0.33 14	−0.56 17
**p75NTR**	0.22 26	0.49* 26	0.54* 26	0.61** 26	0.53* 26	0.41 25	−0.62** 27
**UCHL1**	−0.57*** 42	−0.65**** 42	−0.55*** 42	−0.75**** 42	−0.73**** 42	−0.49** 35	0.81**** 45

Spearman’s rank correlation analyses showing the strength of the association (*r*, upper value), sample size number (n, lower value), and adjusted P values with Holm-Šídák multiple-comparison correction method (adjusted for each retinal neuronal marker against 7 brain pathology and cognitive scores):

* P < 0.05,

** P < 0.01,

*** P < 0.001,

**** P < 0.0001.

**Table:** Key resources table

*Primary antibody*	Source Species	Dilution	Application	Commercial Source	Catalog Number
PSD95 mAb	Rabbit	1:600	IF/WB/TEM	abcam	ab76115 RRID: AB_1310620
VGLUT1 pAb	Guinea Pig	1:1000	IF/TEM	EMD Millipore	AB5905 RRID: AB_2301751
Synaptophysin mAb	Mouse	1:1000	IF/WB	Biolegend	837101 RRID: AB_2565370
NMDAR2A pAb	Rabbit	1:1000	IF	NovusBio	NB300-105 RRID: AB_10001400
p75NGFR (p75NTR) mAb	Rabbit	1:100	IF/WB	abcam	ab52987 RRID: AB_881682
UCHL-1 (PGP9.5) mAb	Rabbit	1:1250	IF/DAB/WB	abcam	ab108986 RRID: AB_10891773
12F4 (Aβ_42_) mAb	Mouse	1:200	IF	Biolegend	805503 RRID: AB_2564682
scFvA13 (Oligo-Aβ) mAb	Mouse*	1:450	IF	Dr. Giovanni Meli Lab	−
Ser396 (pS396-tau) pAb	Rabbit	1:1500	IF	Anaspec	AS-54977
AT8 (Ser202/Thr205) phospho-tau) mAb	Mouse	1:250	IF	ThermoFisher	MN1020 RRID: AB_223647
T22 (Oligo-tau) pAb	Rabbit	1:200	IF	Dr. Rakez Kayed Lab	−
CitR_209_-tau	Mouse	1:5000	IF	Daniel Lee Lab	−
PHF-1(Ser396/Ser404) phospho-tau mAb	Mouse	1:200	IF	Dr. Peter Davies Lab	−
MC-1 (NFT) mAb	Mouse	1:200	IF	Dr. Peter Davies Lab	−
Vimentin mAb	Mouse	1:200	IF	abcam	ab28028 RRID:AB 778826
GAPDH (D16H11) mAb	Rabbit	1:1000	WB	Cell signaling	5174 RRID: AB_10622025
GAPDH mAb	Mouse	1:1000	WB	Millipore Sigma	G8795 RRID: AB_1078991
***Secondary antibody***					
Cy2 (anti- mouse, Rabbit)	Donkey	1:200	IF	Jackson ImmunoResearch Laboratories	
Cy3 (anti- mouse, Guinea pig, Rabbit)	Donkey	1:200	IF		
Cy5 (anti- mouse, Guinea pig, Rabbit)	Donkey	1:200	IF		
IRDye^®^ 680RD pAb	Rabbit	1:10000	WB	Licorbio	926-68071 RRID: AB_10956166
IRDye^®^ 800CW pAb	Rabbit	1:10000	WB	Licorbio	926-32211 RRID: AB_621843
IRDye^®^ 680RD pAb	Mouse	1:10000	WB	Licorbio	926-68070 RRID: AB_10956588
IRDye^®^ 800CW pAb	Mouse	1:10000	WB	Licorbio	926-32210 RRID: AB_621842
HRP anti-rabbit	Goat	1:10000	WB	Vectastain^®^ Elite ABC-HRP kit	PK-6101
***Key reagents and kits***					
Cresyl Violet acetate (Nissl stain)	−	0.1%	Histology	Sigma-Aldrich	C5042
Dihydroethidium (DHE)	−	5–10μM	Histology	Sigma-Aldrich	D7008
Vectastain Elite ABC HRP kit	−	−	Histology	Vector	PK-6101
3,3′-diaminobenzidine (DAB)	−	−	Histology	DAKO	K3468
3-Aminopropyltriethoxysilane (APES)	−	2%	Slide coating	Sigma-Aldrich	A3648
Fluoromount-G, with DAPI	−	−	Histology	ThermoFisher	00-4959-52
Human Amyloid beta 42 ELISA Kit	−	−	ELISA	ThermoFisher	KHB3441
4–20% precast polyacrylamide gels	−	−	WB	Bio-Rad	4561094
***Software and algorithms***					
GraphPad Prism (Version 10.0.3)	GraphPad				http://www.graphpad.com; RRID: SCR_002798
Zeiss Zen Lite	Zeiss				https://www.zeiss.com/microscopy/us/products/software/zeiss-zen-lite.html
Fiji ImageJ (Version 2.14.0/1.54f/Java 1.8.0-322)	Fiji				open-source download
Image Studio software	Licorbio				https://www.licorbio.com/image-studio
Metascape	https://metascape.org/				open source
Cytoscape 3.10.2	https://cytoscape.org/				open source
ClustVis	https://biit.cs.ut.ee/clustvis/				open source
Ingenuity Pathway Analysis (IPA)	Qiagen				https://digitalinsights.qiagen.com/
SynGO	https://syngopotal.org/				open source
String 12.0	https://string-db.org/				open source
***Deposited data***					
Mass spectrometry dataset	ProteomeXchange Consortium via the PRIDE https://www.ebi.ac.uk/pride/archive/projects/PXD040225 https://ftp.pride.ebi.ac.uk/pride/data/archive/2025/10/PXD040225				PXD040225
Machine learning	Scikit-learn NumPy pandas SciPy Jupyter^128^				https://scikit-learn.org/stable/index.html https://numpy.org/ https://pandas.pydata.org/ https://scipy.org/ https://jupyter.org/
